# Evaluation of SMN Protein, Transcript, and Copy Number in the Biomarkers for Spinal Muscular Atrophy (BforSMA) Clinical Study

**DOI:** 10.1371/journal.pone.0033572

**Published:** 2012-04-27

**Authors:** Thomas O. Crawford, Sergey V. Paushkin, Dione T. Kobayashi, Suzanne J. Forrest, Cynthia L. Joyce, Richard S. Finkel, Petra Kaufmann, Kathryn J. Swoboda, Danilo Tiziano, Rosa Lomastro, Rebecca H. Li, Felicia L. Trachtenberg, Thomas Plasterer, Karen S. Chen, on behalf of the Pilot Study of Biomarkers for Spinal Muscular Atrophy (BforSMA) Trial Group

**Affiliations:** 1 Departments of Neurology and Pediatrics, The Johns Hopkins University, Baltimore, Maryland, United States of America; 2 Spinal Muscular Atrophy Foundation, New York, New York, United States of America; 3 Departments of Neurology and Pediatrics, Children’s Hospital of Philadelphia and University of Pennsylvania School of Medicine, Philadelphia, Pennsylvania, United States of America; 4 Department of Neurology, Columbia University, New York, New York, United States of America; 5 Departments of Neurology and Pediatrics, University of Utah School of Medicine, Salt Lake City, Utah, United States of America; 6 Instituto di Genetica Medica, Università Cattolica S. Cuore, Roma, Italy; 7 New England Research Institutes, Inc., Watertown, Massachusetts, United States of America; 8 Department of Chemistry and Chemical Biology, Northeastern University, Boston, Massachusetts, United States of America; Charité Universitätsmedizin Berlin, NeuroCure Clinical Research Center, Germany

## Abstract

**Background:**

The universal presence of a gene (SMN2) nearly identical to the mutated SMN1 gene responsible for Spinal Muscular Atrophy (SMA) has proved an enticing incentive to therapeutics development. Early disappointments from putative SMN-enhancing agent clinical trials have increased interest in improving the assessment of SMN expression in blood as an early “biomarker” of treatment effect.

**Methods:**

A cross-sectional, single visit, multi-center design assessed SMN transcript and protein in 108 SMA and 22 age and gender-matched healthy control subjects, while motor function was assessed by the Modified Hammersmith Functional Motor Scale (MHFMS). Enrollment selectively targeted a broad range of SMA subjects that would permit maximum power to distinguish the relative influence of SMN2 copy number, SMA type, present motor function, and age.

**Results:**

SMN2 copy number and levels of full-length SMN2 transcripts correlated with SMA type, and like SMN protein levels, were lower in SMA subjects compared to controls. No measure of SMN expression correlated strongly with MHFMS. A key finding is that SMN2 copy number, levels of transcript and protein showed no correlation with each other.

**Conclusion:**

This is a prospective study that uses the most advanced techniques of SMN transcript and protein measurement in a large selectively-recruited cohort of individuals with SMA. There is a relationship between measures of SMN expression in blood and SMA type, but not a strong correlation to motor function as measured by the MHFMS. Low SMN transcript and protein levels in the SMA subjects relative to controls suggest that these measures of SMN in accessible tissues may be amenable to an “early look” for target engagement in clinical trials of putative SMN-enhancing agents. Full length SMN transcript abundance may provide insight into the molecular mechanism of phenotypic variation as a function of SMN2 copy number.

**Trial Registry:**

Clinicaltrials.gov NCT00756821

## Introduction

Spinal muscular atrophy (SMA) is an autosomal recessive neuromuscular disorder that manifests across a wide range of severity. The cardinal clinical feature of SMA – diffuse skeletal muscle weakness – is a consequence of dysfunction or loss of alpha motor neurons. SMA is caused by loss-of-function mutations or deletions of the gene SMN1 (Gene ID = 6606). A wide range of disease severity can be partially attributed to the presence of variable copy number of a neighboring near-identical gene, SMN2 (Gene ID = 6607) [1]–[3]. A single base pair difference between these two genes greatly reduces the efficiency of exon 7 inclusion into mature transcripts from the SMN2 gene, but the coded protein sequence from full length SMN2 derived transcripts is unaffected by this change. SMN2 thus produces only a fraction of the functional full length protein compared to SMN1 [4]. The SMN gene is constitutively expressed in all eukaryotic cells and necessary to cell viability [5]–[8]. Motor neurons are particularly vulnerable to reduced SMN protein levels for reasons unknown. The invariable presence of the partially functional disease-ameliorating SMN2 gene in individuals with SMA offers an attractive target for development of therapeutics [9]. Proof-of-concept studies with small molecule, antisense oligonucleotide or gene therapy approaches targeting various mechanisms to upregulate expression of SMN2 have extended survival and improved motor function in SMN-deficient animal models [10]–[16]. Clinical trials of available putative SMA treatments that non-specifically act on SMN expression have, so far, failed to demonstrate efficacy [17]–[19].

One impediment to efficient trial design is that the typical individual with SMA old enough to cooperate with motor function testing declines very slowly [20]. Many affected children settle into a plateau phase with stable function for years even though they may have manifested progressive weakness in the first year or two of life [21]. The consequence of this course is that a meaningful attenuation of the rate of degeneration would necessarily take a long time to demonstrate. Availability of a valid clinical measure of SMN expression could thus accelerate clinical trials of an SMN-enhancing therapeutic, particularly in the early, dose-finding, phase of development. Given that most new treatments currently under development intend to increase SMN synthesis in the nervous system, measurement of the abundance of SMN transcript or protein in tissues accessible to clinical sampling could be an immediate and plausible biomarker that fulfills the need for an early read-out of target engagement.

The continuous spectrum of SMA phenotype severity is generally divided into three “Types” of SMA based upon the history of specific gross motor abilities achieved before the disease curtailed further developmental progress [22]. “Type I” defines those who never sat independently, “Type II” those who sat but never walked, and “Type III” those who were able to achieve independent ambulation. Over time, an individual having a milder type of SMA may decline to a level that overlaps in function with that manifested at an early point in the course of individuals affected by more severe types. The genomic number of SMN2 copies correlates with SMA types [1], [2]. Severity of motor impairment in SMA is likely multifactorial, although disabling mutation of SMN is the essential first step. The potential broad range of SMA phenotype that is predicted by loss of SMN1 is focused to some extent by characterization of SMN2 copy number, but within each specific SMN2 genotype there is broad spectrum of motor function, and over time the severity of motor impairment can vary even more.

The BforSMA study was designed to explore potential biomarkers of disease severity in accessible peripheral tissues – blood and urine – that may be of value in the execution of clinical trials. The cohort was selectively recruited to represent subjects with a wide range of SMA type, motor function, and age, who were then characterized clinically in a manner that would best power two independent projects. Results of the first project, an unbiased biomarker discovery effort, are presented in a companion paper (see companion paper, Finkel et al. [55]). Here, we present the second project, a targeted analysis of relationships between SMN transcript and protein levels to SMN genotype, SMA clinical type, present motor function, and age. The project had two major goals. The first was to determine whether a previously identified association between SMN2 copy number and disease severity could be confirmed in an SMA cohort prospectively recruited to include individuals having all three types of SMA manifesting a broad range of motor impairment. A second, more focused, goal was to evaluate a possible relationship between SMN transcripts and protein levels in blood samples from SMA subjects compared to controls, and within the range of SMA clinical severity. Relationships between any of these measures of SMN in blood samples and clinical features may, with proper further development, prove invaluable for clinical trials of SMN-enhancing therapeutics.

Important to this project was the recognition that age is an important potential confounder to be included in the statistical analysis. The power of this is enhanced by targeted recruitment of a clinical cohort in which SMA type and age are not highly correlated. At the outset of the study, the extent to which this broad distribution could be accomplished was unknown, given early childhood mortality of subjects with SMA Type I. Site investigators were encouraged to keep this goal in mind during subject recruitment.

To date, studies of SMN transcript and protein levels from amniocytes, skin fibroblasts, lymphocytes or peripheral blood mononuclear cells (PBMCs) from SMA subjects have reported variable results, but a general correlation of transcript or protein quantity with phenotype is identified only in individuals at the lower end of the SMA functional continuum [23]–[27]. Protein levels in tissues from SMA Type I infants were noticeably lower than those of healthy controls, while the differences in protein levels between Types II, III, carriers and control subjects were not significant. Existing studies have not controlled for present level of motor function or subject age, and the possibility that these factors undermine or enhance the relationship of blood assessments of SMN to clinical variables is unexplored. In addition, newly developed SMN transcript measurements by absolute quantification and improved ELISA-based protein assays have the advantage of increased sensitivity and reliability [24]–[26], and may prove helpful in characterizing the relationships between protein and disease severity in various tissues. It is thus not yet clear if blood-derived SMN transcript or protein levels can be used as a sensitive and meaningful marker of disease severity.

## Materials and Methods

### Planning and Objectives

The protocol was developed by a combination of SMA patient advocates, experts in biostatistics and bioinformatics, basic scientists investigating the biology of SMA, drug metabolism and pathway experts, and clinical trial specialists. The protocol supported two related, but independent, objectives: (1) an unbiased protein, metabolite and transcript biomarker discovery analysis, and (2) the SMN-targeted biomarker analysis reported here, which intends to identify the relationship of quantitative measures of SMN expression to clinical features of SMA. A leading instrument for the measurement of gross motor function in SMA is the Modified Hammersmith Functional Motor Scale (MHFMS), and although this scale was initially designed and validated only for those with Type II SMA, it is suitable also to evaluate Type III individuals who have lost independent ambulation and to a lesser extent to some of those with only weak ability to walk [28]. It is not suitable for evaluation of many with SMA III. The MHFMS was used to assess all SMA Type II and III and control subjects. Subjects with Type I SMA were assigned a Hammersmith value of zero.

### Study Design

A cross-sectional, single visit, multi-center, exploratory study design was employed. Blood for SMN transcript and protein analysis and SMN2 copy number determination was collected. No therapeutic intervention occurred. There was special emphasis on enrolling children as subjects across a range phenotype severity. The full protocol is available in the Appendix S1.

### Study Management

The administration and data coordination of the trial was conducted by the New England Research Institutes (NERI, Watertown, MA). A protocol committee was established to design the protocol and review potential violations as the trial progressed. Eighteen academic clinical sites headed by investigators with special expertise in the care of children with SMA participated in the study. A meeting of investigators and clinical evaluators from each study site was held to introduce them to the study protocol. All data entry was performed via a secure internet-based website. Sample collection, handling and chain of custody procedures were designed to ensure the best quality specimens for biomarker analysis. Samples were divided for specific analysis and shipped from study sites to the central lab, PPD, Inc. (Highland Heights, KT.) SMN transcripts were measured by Dr. F. Danilo Tiziano laboratory (Istituto di Genetica Medica, Università Cattolica S. Cuore, Roma, Italy), SMN2 copy number from blood were analyzed by Expression Analysis, Inc. (Durham, NC), and SMN protein analysis by an ELISA assay developed and run at Enzo Life Sciences (Ann Arbor, MI). The Project Director and Data Management group at NERI was responsible for data processing while their Biostatistics group handled the data analyses.

### Ethics Statement

Institutional review board (IRB) approval for the protocol was obtained from each BforSMA clinical site before enrollment at that site and from the central Institutional Review Board, New England Research Institutes. Written informed consent for participation was obtained from the legal guardians of all subjects and assent for participation was obtained directly from subjects whenever applicable. This trial was registered with ClinicalTrials.gov with identifier NCT00756821.

### Sample Size Determination and Enrollment

The number and type of subjects enrolled was based upon the expected power necessary for the unbiased discovery project. The sample size had an 80% power at level 0.05 to detect an effect size of 0.6 standard deviations between SMA and controls, which is considered a moderate difference. An unbalanced number of subjects from the three types of SMA for targeted enrollment was selected (15 Type I, 45 Type II, and 40 Type III SMA subjects) in part due to the expectation that recruitment of eligible subjects with SMA Type I across the full age range would be difficult due to the high level of mortality in childhood [29].

### Inclusion and Exclusion Criteria

The ideal population would enable independent assessment of the contribution of SMN genotype, SMA type, present functional status, and age in biomarker characterization. The age range chosen excluded subjects less than 2 years of age – to avoid metabolic confounders of infancy, nascent developmental accomplishments, and incomplete cooperation with the functional outcome measure – and also excluded those over 12 years of age to limit the contribution of puberty-related confounders [28]. Additional eligibility criteria highly restricted the use of potential disease-modifying prescription medication and excluded children who were affected by acute diseases, unrelated to SMA. SMA subjects were required to have genetic confirmation of disabling mutation of SMN1 and were defined into standard categories of SMA by their history of ability (Type II) or inability (Type I) ever to sit unaided for 30 seconds, or ability to ever stand and walk 30 feet unaided (Type III) [22]. To limit biased ascertainment of individuals into the healthy control cohort that might shift the proportion of single-copy SMN1 genomes from that of the normal population, only those SMA siblings known by previous genetic evaluation to have 2 or more copies of SMN1 were eligible. Initial caps on the maximum number of subjects enrolled per site were instituted to enhance broad applicability of results.

### Statistical Analysis for SMN Copy Number, Transcript and Protein

Potential predictors of SMN protein, transcript and copy numbers included: age, gender, SMA diagnosis and type, and current level of function as measured by the MHFMS. Relationships among SMN protein, individual transcript types (listed below), and copy numbers were examined as well. One extreme outlier of SMN transcript ratios (SMN2-FL/SMN-Δ7 ratio, 26 standard deviations above the next highest value) was removed from analysis of that outcome alone. Predictors of continuous outcomes were assessed using analysis of covariance (ANCOVA) for categorical predictors and partial correlation for continuous predictors. As a significant effect of age was found for some outcomes, all models were controlled for age. Data was log-transformed to reduce the impact of skewed data. All analyses were performed with SAS v9.2 (SAS Institute, Cary, NC), and p<0.05 was considered statistically significant. By design, our study cohort included subjects whose function fell outside the range of MHFMS detection, and thus were scored the constrained maximum or minimum MHFMS value. Because ceiling and floor values will increase (positive) or decrease (negative) slopes of identified associations we performed the statistical analysis of SMN transcripts and protein to MHFMS comparisons by including and excluding those with border values.

### SMN Copy Number

Quantification of SMN1 and SMN2 copy number was conducted at Expression Analysis Inc., Durham, North Carolina by quantitative real-time Taqman PCR (qPCR) [30]. Genomic DNA was isolated from whole blood samples using Gentra Puregene Blood Kit (Qiagen). Externally validated DNA standards for SMN1 and SMN2 were kindly provided by Dr. Wendy Chung from Columbia University Medical Center, New York, NY. The SMN1 and SMN2 reactions were carried out in 1×TaqMan Universal PCR master mix (Applied Biosystems) containing 300 nM of SMN1 primers, 250 nM of SMN probe or 450 nM of SMN2 primers, 250 nM of SMN probe, 650 nM of SMN1 non-extending oligonucleotide, respectively. The non-extending SMN1 oligonucleotide increases SMN2 assay specificity by blocking nonspecific annealing of the allele-specific primer to the opposite allele. To enable normalization of the input target DNA added to each well, the internal control CFTR gene was amplified simultaneously in a separate reaction well under identical thermal cycling conditions. The CFTR reaction was carried out with 1×TaqMan Universal PCR master mix containing 450 nM of CFTR primers and 250 nM of CFTR probe. PCR was performed on a 7900HT Sequence Detection System (Applied Biosystems) using a 384-well format, and amplification was achieved using the standard amplification protocol (Applied Biosystems) as follows: 50°C for 2 min, 95°C for 10 min, followed by 45 cycles of 95°C for 15 s, and 60°C for 1 min. Each reaction was run in quadruplicate with 25 ng of genomic DNA in a final volume of 15 µl. The number of SMN1 and SMN2 copies was calculated using the comparative C_T_ method [31]. Results were interpreted separately by two independent investigators (Dr. Wendy Chung, Columbia University Medical Center, NY, NY; and Dr. Louise Simard, University of Manitoba, Winnipeg, Manitoba) and confirmed in a separate analysis performed by Dr. Thomas Prior at The Ohio State University using his published methodology [27].

### SMN Transcripts

Four specific transcripts were measured: (1) SMN2-Full Length (SMN2-FL), (2) SMN1-Full Length (SMN1-FL), (3) SMN transcript lacking exon 7 (SMN-Δ7), and (4) GAPDH, a housekeeping gene commonly used for normalization in SMN transcript analysis. Three combinations of these primary measurements were also considered of potential relevance: (5) Total SMN-FL (SMN1-FL+SMN2-FL), (6) Total SMN (SMN-FL+SMN-Δ7), and (7) a ratio of SMN2 transcripts that reflects exon 7 inclusion (SMN2-FL/SMN-Δ7). As SMN1-FL is only found in control subjects, the values of SMN2-FL and Total SMN-FL are the same in SMA subjects.

SMN2-FL and SMN1-FL levels in whole blood were evaluated by absolute real-time PCR [26]. GAPDH transcript levels were determined as positive controls both for reverse transcriptase PCR (RT-PCR) and real-time PCR in similar fashion. Analysis of SMN-Δ7 transcripts followed a procedure similar to that of SMN1-FL and SMN2-FL transcript quantification using primers SMN-Δ7-absF CTG ATG CTT TGG GAA GTA TGT TAA TT and SMN-Δ7-absR CCA GCA TTT CCA TAT AAT AGC CAG TA, and probe SMN-Δ7_absP 5′FAM - CAT GGT ACA TGA GTG GCT A -NFQ3′.

### SMN Protein

A total of 127 samples (SMA 105, control 22) were available for SMN ELISA measurements. Whole blood was collected into EDTA K_2_ tubes and approximately 4 mL was poured into CPT vacutainers (#362760 BD, Franklin Lakes, NJ). PBMCs were isolated by centrifugation at 1500 rpm within 2 hours of collection. PBMC samples were shipped at ambient temperature to PPD for further processing and frozen storage. Frozen samples were transferred to Enzo Life Sciences (Ann Arbor, MI) for SMN analysis. PBMCs were thawed in a 37°C water bath and viable cell hemocytometer counts, performed immediately prior to lysis, were used to determine the appropriate volume of lysis buffer (LB-11) needed to establish a consistent concentration of the cell suspension of 10^8^ cells per milliliter. LB-11 containing 300 mM NaCl, 10% glycerol, 3 mM EDTA, 1 mM MgCl_2_, 20 mM b-glycerophosphate, 25 mM NaF and 1% Triton X-100 was used along with protease inhibitors PIC8340 (Sigma #P8340, St. Louis, MO) and phenylmethylsulphonyl fluoride (Sigma #P7626). The cell suspension was gently vortexed and placed on ice for 30 minutes. The cell lysate was transferred to a 1.5 mL centrifuge tube and was clarified by centrifugation for 10 minutes at 14,000 RCF, 4°C. The supernatant was transferred to a clean vial and either assayed immediately or stored at –70°C until use.

Recombinant human SMN1 was generated from full-length cDNA expressed in bacterial expression vectors and purified for use as a standard in the ELISA. The capture antibody Sigma anti-SMN clone 2B1 (#S2944) was coated at 100 uL onto Costar Stripwell (#92592, Lowell, MA) at 3.5 ug/mL. After overnight incubation at room temperature, the plate was blocked for 5 hours with 1% BSA in PBS. Cell lysate samples and recombinant hSMN1 or HeLa cell lysate standards were loaded at 100 uL per well. Standards were diluted in 2-fold dilutions or from 0.0625-4 ng/mL. Samples were incubated for one hour at room temperature, washed and then incubated with a detection antibody from ProteinTech (#11708-AP-1, Chicago, IL) at 2 ug/mL for one hour at room temperature. After washing, a peroxidase conjugated goat anti-rabbit IgG from Jackson Immunolabs (5-#035-144, West Grove, PA) was applied at 50 ng/mL to the plate and incubated for 30 minutes at room temperature. After washing, plates were developed with TMB substrate for 30 minutes at room temperature and the reaction stopped after 30 minutes with 1N HCl acid. Plates were then read on a spectrophotometer at 450 nm. Plates were sealed and gently shaken during all incubations, and dilutions of sample and standard were done in assay buffer (1% BSA, 0.1% Tween-20 in PBS).

## Results

### Characteristics of the Study Cohort

**Figure 1:**
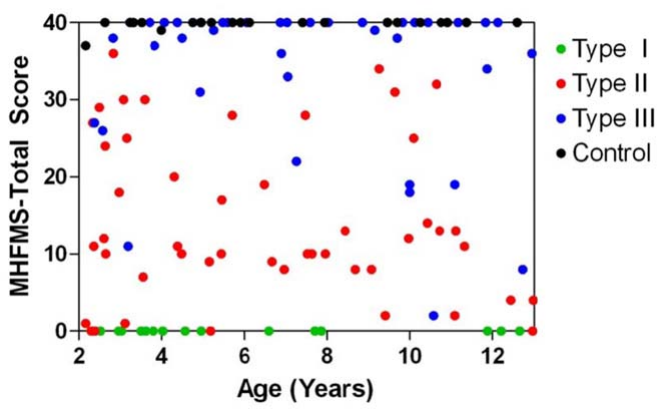
Modified Hammersmith Functional Motor Scale versus age by SMA cohort. Scores for the MHFMS were well-distributed by age across the enrollment cohorts. It should be noted that not all control individuals achieved a score of 40 on the scale, while all Type I SMA subjects were assigned scores of zero in the assessment.

A total of 130 subjects were enrolled, including 17 Type I, 49 Type II, 42 Type III SMA, and 22 healthy control subjects. Enrollment figures slightly exceeded the original targets for each group due to replacement, per protocol, of subjects in whom specimens were of insufficient quantity (n = 7) or quality (n = 3). All 18 study sites contributed at least one subject over the course of 18 weeks concluding in March 2009. The age and gender distribution of those enrolled is presented in the second table of companion paper, Finkel et al. [55] Although age of onset correlates with phenotype severity in SMA, a key recruitment goal of this study was to minimize within the ascertained cohort the correlation between present age and present functional status – because such correlation might introduce an age bias into identified markers and reduce the power of discrimination between factors associated with SMN2 copy number, SMA type, and motor function. This goal was achieved both overall and within SMA groups Type II and III, and to a partial extent, SMA Type I (Figure 1, Table 1, Figure S1 and Table S1, see companion paper, Finkel et al. [55]).

**Table 1:** SMA and Control Subjects’ Clinical Data.

	SMA Type IN = 17	SMA Type IIN = 49	SMA Type IIIN = 42	ControlN = 22	p-value*Type I vs II vs III	p-value* SMA vs Control
**Age (years)**					0.14	0.84
mean (SD)	5.70 (3.54)	6.55 (3.40)	7.51 (3.11)	6.95 (3.29)		
median [range]	4.03 [2.39–12.66]	6.49 [2.16–12.98]	7.42[2.37–12.95]	6.02 [2.16–12.59]		
**Sex,** n (%)					0.73	0.64
male	10 (58.8%)	26 (53.1%)	20 (47.6%)	10 (45.5%)		
female	7 (41.2)	23 (46.9%)	22 (52.4%)	12 (54.6%)		
**MHFMS**					<0.001	<0.001
mean (SD)	0 (0)	14.02 (10.55)	34.1 (10.0)	39.8 (0.7)		
median [range]	0 [0–0]	11 [0–36]	40 [2]–[40]	40 [37]–[40]		

* ANOVA for continuous variables; Fisher exact test for categorical variables.

Legend: There were no significant differences in age or gender across the recruitment cohorts. The Modified Hammersmith Motor Function Scale differentiated between SMA subjects and controls and between Type I, II and III subjects.

### SMN2 Copy Number

**Figure 2:**
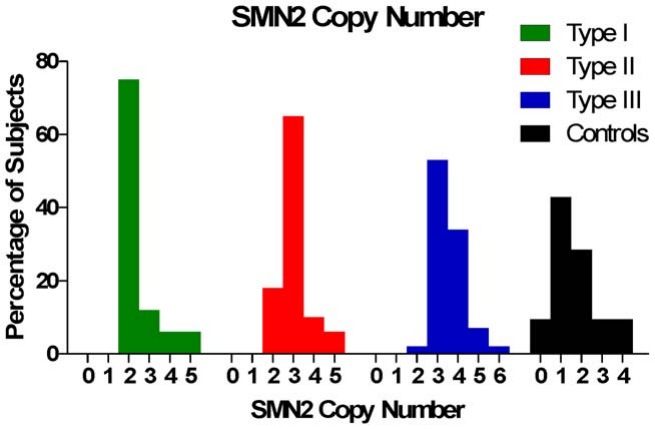
SMN2 copy numbers in SMA and Control subjects. SMN2 copy number is lower in Controls than it is in SMA subjects, in whom copy number is related to type.

Copy number of SMN2 varied in the SMA subjects from 1 to 6, and distributed with SMA type in a manner consistent with previous experience [1], [2], [32], confirming the hypothesis that there is an inverse relationship between SMA severity and SMN2 copy number in this cohort (Figure 2). SMN2 copy number increased and was strongly associated (p = 0.002) with SMA type (Figure 2 and Table S2). SMA Types I and II most commonly had 2 and 3 copies of SMN2 respectively, and Type III SMA had 3 or 4 copies of SMN2 (Table 2). This SMA cohort included a few prominent outliers of the trend correlating SMA type to SMN2 copy number including Type 1 subjects with high SMN2 copy number, and Type III SMA subjects with a low SMN2 copy number. Additionally, it was noteworthy that this cohort contained SMA subjects with 5 or 6 copies of SMN2.

**Table 2:** SMA and Controls SMN Transcripts, Protein and Copy Number.

	SMA Type I	SMA Type II	SMA Type III	Control
**SMN2 Copy Number**	**N = 16**	**N = 48**	**N = 42**	**N = 21**
mean (SD)	2.5 (0.9)	3.0 (0.7)	3.5 (0.8)	1.7 (1.1)
median [range]	2.1 [1.8–5.2]	2.9 [1.8–5.2]	3.2 [2.2–5.9]	1.3 [0.0–4.2]
**SMN Protein (pg/10^6^ cells)**	**N = 13**	**N = 43**	**N = 38**	**N = 22**
mean (SD)	10.1 (4.8)	14.0 (7.3)	14.7 (10.0)	25.2 (19.6)
median [range]	10.6 [2.3–19.4]	12.8 [1.6–33.1]	12.4 [3.1–45.8]	18.8 [5.0–86.2]
**SMN2-FL Transcripts (mol/ng)**	**N = 13**	**N = 36**	**N = 31**	**N = 13**
mean (SD)	38.3 (16.3)	49.0 (18.6)	58.1 (20.0)	51.5 (22.3)
median [range]	32.1 [19.1–70.2]	48.9 [17.3–94.8]	54.2 [31.8–111.2]	55.6 [15.5–91.7]
**SMN-FL Transcripts (mol/ng)**	**N = 13**	**N = 36**	**N = 31**	**N = 13**
mean (SD)	38.3 (16.3)	49.0 (18.6)	58.4 (19.7)	134.1 (65.1)
median [range]	32.1 [19.1–70.2]	48.9 [17.3–94.8]	54.2 [34.0–111.2]	129.9 [40.9–259.8]
**SMN-Δ7 Transcripts (mol/ng)**	**N = 13**	**N = 36**	**N = 31**	**N = 13**
mean (SD)	140.6 (69.8)	202.6 (73.0)	200.4 (97.5)	115.2 (64.2)
median [range]	133.5 [65.5–320.8]	212.0 [54.4–316.9]	188.9 [52.8–400.0]	121.3 [16.2–236.9]
**SMN-total Transcripts (mol/ng)**	**N = 13**	**N = 36**	**N = 31**	**N = 13**
mean (SD)	178.8 (76.5)	251.6 (82.1)	258.8 (110.5)	249.4 (98.3)
median [range]	163.1 [95.7–361.3]	278.2 [78.3–367.8]	247.4 [95.5–475.4]	267.6 [63.9–448.8]
**SMN2-FL/SMN-Δ7 Transcripts**	**N = 13**	**N = 36**	**N = 31**	**N = 13**
mean (SD)	0.31 (0.13)	0.26 (0.09)	34.1 (0.16)	0.44 (0.15)
median [range]	0.33 [0.13–0.52]	0.27 [0.09–0.45]	0.28 [0.14–0.81]	0.42 [0.22–0.68]
**GAPDH Transcripts (mol/ng)**	**N = 12**	**N = 36**	**N = 30**	**N = 13**
mean (SD)	4937.0 (1900.1)	3662.0 (1870.4)	3165.1 (757.5)	4251.5 (2079.1)
median [range]	4535.0 [2428.6–8180.0]	3298.5 [1450.0–7860.0]	3054.5 [1580.0–4520.0]	3570.0 [2230.0–9330.0]

Mean and median values of SMN transcripts, protein and copy number for SMA Type I, II, III and Controls. Transcript values are presented as number of molecules per ng of total RNA (mol/ng).

Deviations from the usual relationship between copy number and SMA type further confirm that SMN2 copy number alone does not predict individual phenotype severity. SMN2 copy number was significantly lower in control subjects (p<0.001), with values similar to other reports [1], [33]. This trend, previously reported, likely relates to two processes: (1) embryonic lethality of genomes containing 0 copies of SMN1 and less than 2 copies of SMN2, and (2) pathogenic SMA alleles often contain a conversion type mutation wherein the sequence of SMN1 is converted to that of SMN2, increasing SMN2 copy number in individuals with the less severe forms of SMA [34]–[36].

**Figure 3:**
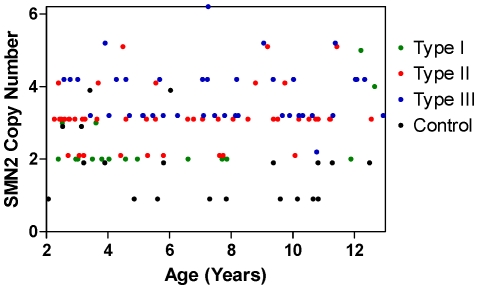
SMN2 copy number relationship to age, and by SMA Type or Control. Subjects with each SMA type are broadly distributed across the age range, with the exception of type I SMA for whom there is some bias to younger age. As a consequence, SMN2 copy numbers are also broadly distributed. Values have been plotted with a small y-axis offset to avoid overlap of values.

Figure 3 shows the lack of a relationship between SMN2 copy number and age, whether overall or by type, confirming the success of the recruitment strategy to establish a cohort in which SMA type and age were not correlated. In addition, there was no correlation between SMN2 copy number and MHFMS score in Type II or III SMA groups in isolation, or in the Type II+III group combined, when subjects with maximum or minimum MHFMS scores were removed (data not shown) (21 subjects with a minimum score, and 42 subjects –22 with SMA and 20 control – with a maximum score). There was no association between SMN2 copy number and gender. A post-hoc analysis determined that exclusion of outlier subjects with high copy number did not result in a meaningful change in any of the identified relationships.

### SMN Transcripts, Age and Gender Comparisons

In a regression model controlling for SMA diagnosis and type, SMN2-FL (p = 0.007), SMN-FL (p = 0.008) and SMN-total (p = 0.036) transcript levels decrease with age. SMN-Δ7 and GAPDH transcript levels did not change significantly with age (Figure S1 and Table S1). There was no significant effect of gender on any of the transcript measurements.

### SMN Transcripts: Relationship to SMA Type, SMN Copy Number, and MHFMS

**Figure 4:**
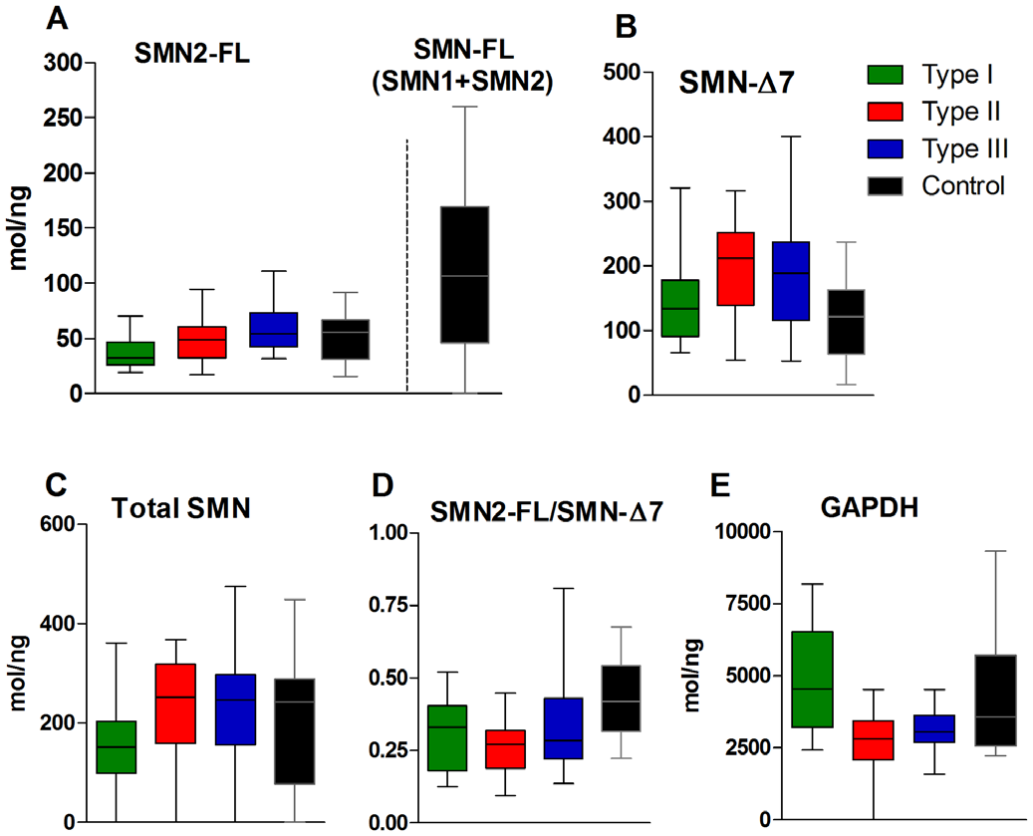
SMN transcript concentrations in SMA and Control subjects. A,C: SMN2-FL, SMN-FL and Total SMN transcripts generally increase with SMA Type. SMN-FL (A) is a sum of SMN1-FL (present only in healthy controls) and SMN2-FL. B: SMN-Δ7 expression levels are lower in Type I patients compared to other SMA Types but they are similar to that of Controls. D: Ratios of SMN2-FL to SMN- Δ7 differ between SMA Types and Controls, however differences between SMA Types are absent with the exception of Type II versus Type III patients. E: GAPDH transcript levels are elevated in SMA Type I and Controls relative to Type II and III patients.

The relationship of SMN transcripts to the three SMA types and control groups is presented in Figure 4 and Table S2. SMN-FL transcript levels increased with SMA type (Figure 4A, Table 2), and levels in all three types were less than that found in controls, due to the presence of SMN1. SMN2-FL transcript level increased with SMA type (Figure 4A, Table 2), but it was similar for Type III subjects and controls. As hypothesized, the analysis of subjects with SMA revealed a significant difference for SMN2-FL (and hence, Total SMN-FL) transcript levels for Type I vs Type II, Type I vs Type III and Type II vs Type III (p = 0.031, <0.001, 0.024, respectively). SMN-Δ7 transcript level was higher in Type II and III compared to Type I and controls (Figure 4B, Table 2). It is notable that the ratio of SMN2-FL/SMN-Δ7 was similar within SMA groups (Figure 4D, Table 2), and is higher in controls, which was mostly driven by lower levels of SMN-Δ7 in controls. Surprisingly, GAPDH transcript levels were significantly different in SMA Type I versus Type II or III subjects (Figure 4E, Table 2).

A noteworthy finding is that within the SMA subject group there was no association of SMN2-FL, (Total SMN-FL) or any other transcript levels to SMN2 copy number. The only significant association between any SMN transcript and SMN2 copy number is that of SMN-Δ7 assessed in the whole study group combining SMA and control subjects (r = 0.31, p = 0.003).

SMN2-FL and SMN-Δ7 transcript levels in SMA subjects showed some modest relationships to MHFMS score when the transcripts were assessed in all SMA subjects, including those with a maximum or minimum MHFMS score value (r = 0.34, p = 0.009 for Type II+III only for SMN2-FL; r = 0.60, p = 0.0001 for Type III only for SMN-Δ7). When the analysis was confined to the 67 subjects having non-maximum or minimum MHFMS scores – to address the possible confounders associated with floor and ceiling effects – the relationship between SMN2-FL and SMN-Δ7 transcripts and MHFMS was lost.

### SMN Protein

**Figure 5:**
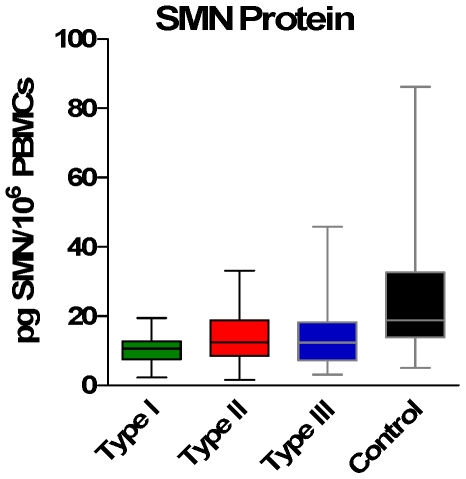
SMN protein levels in SMA and Control Subjects. While SMN protein levels are lower in SMA relative to Control subjects, protein levels by SMA type are not statistically different from each other.

As expected, SMN protein was significantly lower in SMA samples compared to control (Figure 5). Counter to our expectations, SMN protein levels did not differentiate between types (mean of 14.1, 14.0 and 14.7 pg/10^6^ PBMCs for Types I, II and III; Table 2). SMN protein levels did correlate with SMN2 copy number for all SMA subjects as one group (r = 0.33, p = 0.001), with the largest contribution of that relationship from Type II SMA subjects (r = 0.41, p = 0.008). SMN protein also correlated with total SMN-FL transcript when all SMA subjects are combined with controls (r = 0.26, p = 0.021). In addition, SMN protein did not correlate with MHFMS.

## Discussion

### Blood-Derived Measures of SMN

This is the first report in which SMN2 copy number, and transcript and protein measures of SMN expression in blood using the quantitative assay methodologies that reflect the current consensus in the field, have been applied prospectively to a large and broad cohort of subjects with SMA. Tables 3 and 4 summarize qualitatively the many comparisons made in this study between measurements of SMN copy number, SMN transcripts, and SMN protein to the clinical variables of MHFMS, SMA type, and SMA diagnosis to healthy controls; and the relationship of the several SMN measures to one another.

**Table 3:** Summary of SMN associations: Pairwise analysis between various SMA groups.

	SMA group vs. control	Between SMA types
**SMN2 copy number**	Yes	Yes
**SMN2-FL transcript**	No	Yes
**SMN protein**	Yes	No

The “Between SMA types” column includes the following comparisons: Type I vs Types II+III, Type I vs Type II, Type I vs Type III, Type II vs Type III.

**Table 4:** Associations between SMN2 copy number, SMN2 full-length transcript, SMN protein and MHFMS.

	SMN2 copynumber	SMN2-FLtranscript	MHFMS
**SMN2 copy number**	N/A	No	No
**SMN2-FL transcript**	No	N/A	No
**SMN protein**	Yes*	No	No

The “SMN2 copy number” and “SMN-FL transcript” columns include the partial correlation analysis for SMA+Controls, SMA (all SMA patients), Type I, Type II, Type III, Controls. The “MHFMS” column includes the analysis for Type II, Type III and Type II+Type III with exclusion from the analysis of subjects having minimum or maximum scores.

* Correlation was only found for the SMA group (r = 0.33, p = 0.001) and Type II group (r = 0.41, p = 0.008).

Our findings confirm the data from previous blood sample studies that show lower SMN-FL transcripts and protein in the most severely affected subjects with SMA Type I [23]–[26]. In addition, this study extends these findings to identify relationships in milder types of SMA, confirming the findings of Tiziano et al. [26] in a smaller cohort. GAPDH transcript levels were statistically different between SMA Type I versus Type II and III patients, suggesting a need for caution in the selection of housekeeping genes for transcript normalization. This lends further support to the use of methods based on absolute standard curves for transcript analysis in order to avoid biases in data interpretation.

There was no relationship between any measure of SMN expression and motor function as measured by the MHFMS within the subset of subjects having non-maximum or non-minimum scores. One important caveat to these findings is that this functional scale was designed for children with SMA Type II. In this study a MHFMS score was given to all subjects (SMA Type I assigned a score of “0”), but only subjects with SMA Type II and those with Type III SMA who are no longer able to walk achieved scores that were inside the minimum and maximum boundary values of the scale. There is, at present, no validated instrument of functional motor assessment that encompasses the entire range of SMA into a single scale. Comparisons of blood derived measures of SMN expression to subject motor function were thus necessarily constrained to a subgroup of subjects. Previous findings [26] of an association of SMN transcript levels to MHFMS may be due to small sample size of the cohort, to the inclusion of maximum and minimum MHFMS value patients, and stronger correlations between SMA type, age, and MHFMS within that cohort compared to this study selectively recruited to minimize these confounders. It is possible that significant associations of blood measures of SMN expression might be possible for other assessments of motor function that target the ranges above or below that assessed by the MHFMS. One consideration for future work is to extend the analysis made here for MHFMS to additional functional tests that better evaluate patients with higher and lower function, as well as do a longitudinal assessment of SMA patients across functional groups.

SMN2 copy number is grossly related to the disease severity but has only modest predictive value for individual patients. SMN transcript levels were reduced in patients compared to controls in a manner graded by SMA type. The differences in SMN-FL abundance relative to SMA type (Figure 4) are modest when compared to the more substantial differences in SMN-FL comparing SMA as a group to healthy controls. This finding supports a hypothesis derived from the genetic epidemiology of SMA, that the differences in SMN expression capacity between SMA types are relatively small. Embryos inheriting only a single copy of SMN2 are subject to early lethality. The differences in clinical expression between those with 2 and 3 copies SMN ranges from early death to easy ambulation, indicating a substantial role of small differences in SMN production capacity. These small differences are apparent in the small group differences found in SMN-FL concentration between SMA Types I, II, and III. Given the wide overlap for SMN transcript levels among the different SMA types, these measures may not have prognostic value as stand-alone measures. Determination of their ultimate predictive value thus requires further exploration, and in particular re-examination in the context of treatment with SMN-targeting drugs.

A notable finding is that SMN2-FL (Total SMN-FL) transcript abundance correlates with SMA type, but not with SMN2 copy number, but SMA type and SMN2 copy number are themselves highly correlated. A similar lack of correlation between SMN transcript and SMN2 copy number was found previously [26], though the smaller cohort size in that study limited the impact of the observation. The fact that SMN transcript is more powerfully associated with SMA type than with SMN2 copy number could identify a molecular basis for the lack of a tight prediction of SMA type by SMN2 copy number. The absence of correlation between SMN2 copy number and SMN2-FL transcript level may be the consequence of (1) differences in regulation of transcription and alternative splicing of individual SMN2 genes, (2) the presence of non-intact variants of the gene, (3) variability in stability of SMN2 transcripts, (4) differences at the genomic level among SMN2 genes [37], (5) high variance in SMN levels or any combination of these factors. These factors might also contribute to additional variability in SMN2-FL levels among SMA patients.

The lack of strong correlation between SMN protein levels, and SMN2 copy number or transcript levels in SMA patients suggests that there may be significant modulation of SMN at the posttranscriptional level. This regulation could be different for different cell types and tissues and it may also vary by age. Moreover, it should be pointed out that PBMCs may not fully reflect SMN expression levels in more disease relevant cells like motor neurons, muscle, or both. There is a large body of evidence that suggests that other genetic modifiers of SMN can significantly impact SMA phenotype in animal models and humans [38]–[41]. In addition, the inclusion or exclusion of exon 7 into SMN2 transcripts is controlled by several protein factors that could contribute to the levels of full-length transcripts [42]–[54]. It is similarly understood that the functional outcome measures assessed in this study are also subject to a wide range of factors. Nevertheless, these results confirm that SMN transcript and protein are measureable in blood, that there is important inter-patient variability in transcript and protein levels, and that neither transcript, protein nor SMN2 copy number are solely predictive of phenotype. It will be critical to explore changes in intra-patient transcript and protein levels over time and in other tissues and the regulatory factors governing transcription and translation.

The absence of an association of SMN transcripts or protein to motor function as assessed by MHFMS might be expected, as SMA likely progresses due to downstream cellular consequences of diminished SMN abundance. This is a robust negative finding, as the power of this study cohort was aided by the successful recruitment of a wide and evenly distributed range of SMA type, SMN genotype, motor function and age. Nevertheless, these findings do not preclude further exploring measures of SMN expression in blood as a biomarker of early pharmacodynamic assessment of *in vivo* “target engagement” of a putative SMN-enhancing agent.

Further studies are also needed to evaluate the potential value of blood-derived measures of SMN expression to SMA therapeutics. The first will be to understand better its performance as a clinical measure. An important extension of this initial cross-sectional analysis will be a longitudinal analysis of individual patients, across the spectrum of SMA type and age, to evaluate the stability of measures of SMN expression over time. The extent to which measures of SMN transcript or protein values in blood are stable in repeated within-subject measurements, as suggested by preliminary findings [26] will determine the power of these assays to evaluate a systemically delivered SMN-enhancing therapeutic agent. A second line of investigation will be to assess the relationship of blood-derived measures of SMN to that of other tissues, and in particular to the expression of SMN in the CNS. To some extent this can be done by comparing SMN transcript and protein in the more difficult, but still accessible, tissues such as skin and muscle, or from CNS tissues available from post-mortem samples of subjects with SMA. Alternatively, comparing blood to CNS-derived measures of SMN expression in relevant animal models may be useful to establish the strength of any relationship between the two. Other future studies that may be valuable to clinical trials, and a better understanding of the biology of SMA and SMN-depletion, would be to identify of the relationship of candidate biomarkers found in the companion study to the markers of SMN abundance identified here.

## Full List of Competing Interests

Dr. Crawford serves on the medical and scientific advisory boards of Families of SMA, the medical board of the Muscular Dystrophy Association, and has served as frequent ad-hoc advisor to the Scientific Advisory Board of the SMA Foundation. He has received research support for clinical studies from Families of SMA, SMA Foundation, and the Ataxia Telangiectasia Children’s Project.; Dr. Paushkin is an employee of the SMA Foundation.; Dr. Kobayashi is an employee of the SMA Foundation.; Ms. Forrest was an employee of the SMA Foundation during the time of the study.; Ms. Joyce was an employee of the SMA Foundation during the time of the study.; Dr. Finkel receives commercial research support from PTC Therapeutics, Santhera Pharmaceuticals, and Genzyme Corp. and non-profit research support from the SMA Foundation and support from the NIH/NIAMS and NIH/NINDS, accepted travel stipends as part of grants from PTC Therapeutics and the SMA Foundation, reviewed and prepared a report for Adibi legal proceeding, spends 50% of his professional time carrying out clinical studies, and serves on the medical advisory board of DuchenneConnect and Families of SMA and on the scientific advisory board of PTC Therapeutics. A member of his immediate family receives commercial research support from Merck Pharmaceuticals, has received license fee payments from Southern Biotechnology Associates, Upstate Pharmaceuticals, and Santa Cruz Biotechnology, receives research funding from the NIH (grant #s: AR058606, 1R21 AI078387, 1R21 AI078387-S1, 1R41 AI071927, R01 AI063623, 1U19AI082726, T32; pending grant #s: 1R21, AR059466, T32, AR059650), holds 6 patents or pending patents, contributes to other clinical research as a local co-investigator or PI in studies funded by the NIH and the UK, is the editor of Janeway Textbook of Immunology and Arthritis Research and Therapy, and devotes 33% of her professional time to clinical studies in her practice.; Dr. Kaufmann is an employee of the federal government and has no disclosures. Prior to August 2009, Dr. Kaufmann was an employee of Columbia University and received research support from the NIH, the SMA Foundation, PTC, Santhera Pennwest and the DoD. This report is based on Dr. Kaufmann’s work at Columbia University and is not related to the National Institutes of Health.; Dr. Swoboda serves on the scientific advisory boards of Families of SMA, the Pediatric Neurotransmitter Disorders Foundation, California Stem Cell, Inc., and the Alternating Hemiplegia of Childhood Foundation (AHCF). She serves as an ad-hoc reviewer for the Muscular Dystrophy Association (MDA) and NIH. She has accepted research funds for consultation for Biomarin Pharmaceuticals and Shire, Inc. She receives or has received in the past year grant funding from Families of SMA, MDA, FightSMA, AHCF and NIH (R01-HD054599; ARRA 5-R01 HD054599-04, and 1-R01-HD69045 from NICHD).; Dr. Tiziano reports no disclosures.; Dr. Lomastro reports no disclosures.; Dr. Li is employed by New England Research Institutes (NERI). NERI was paid by the sponsoring organization of this study to coordinate this study and perform additional analyses.; Dr. Trachtenberg is employed by New England Research Institutes (NERI). NERI was paid by the sponsoring organization of this study to coordinate this study and perform additional analyses.; Dr. Plasterer was employed by BG Medicine (BGM) from July 2001-March 2010. BGM was paid by the sponsoring organization of this study for sample and statistical analysis.; Dr. Chen is an employee of the SMA Foundation.; This does not alter our adherence to all the PLoS ONE policies on sharing data and materials.

## Supporting Information

Figure S1 **Effect of Age on (log) SMN Transcript Levels in SMA and Control Subjects.** Regression of age on SMN-FL (A), SMN-Δ7 (B), SMN-total (C) and GAPDH (D) transcript levels of Type I, II, III and Controls. Transcript values are presented as log of number of molecules per ng of total RNA (mol/ng).
(TIF)

List S1 **Members of the BforSMA Trial Group.**
(DOCX)

Appendix S1 **BforSMA final NERI IRB-approved protocol.**
(DOC)

Table S1 **Effect of Age on (log) SMN Transcript Levels in SMA and Control Subjects.** * Regression of age on SMN transcript levels, controlling for SMA diagnosis and Type. There were no significant interactions between age and diagnosis/type.
(DOC)

Table S2 **SMN Transcripts (log) and Protein (log) by SMA and Control Subjects: pair-wise comparisons.** Analysis of covariance (ANCOVA) of SMA Type I vs. II vs. III vs. Control, controlling for age. The p-values presented here represent pair-wise post-hoc comparisons from the single model. GAPDH values are not shown as they remained unchanged. The mean and median values for transcript, protein and copy number data by SMA type are presented in Table 2. *SMN2 copy number values were not log transformed as they are ordinal values.
(DOC)
