# Supplementary figures and images for: Evaluation of SMN Protein, Transcript, and Copy Number in the Biomarkers for Spinal Muscular Atrophy (BforSMA) Clinical Study
Source: PLoS One. 2012 Apr 27;7(4):e33572. doi: 10.1371/journal.pone.0033572 (PMC3338744; doi:10.1371/journal.pone.0033572)

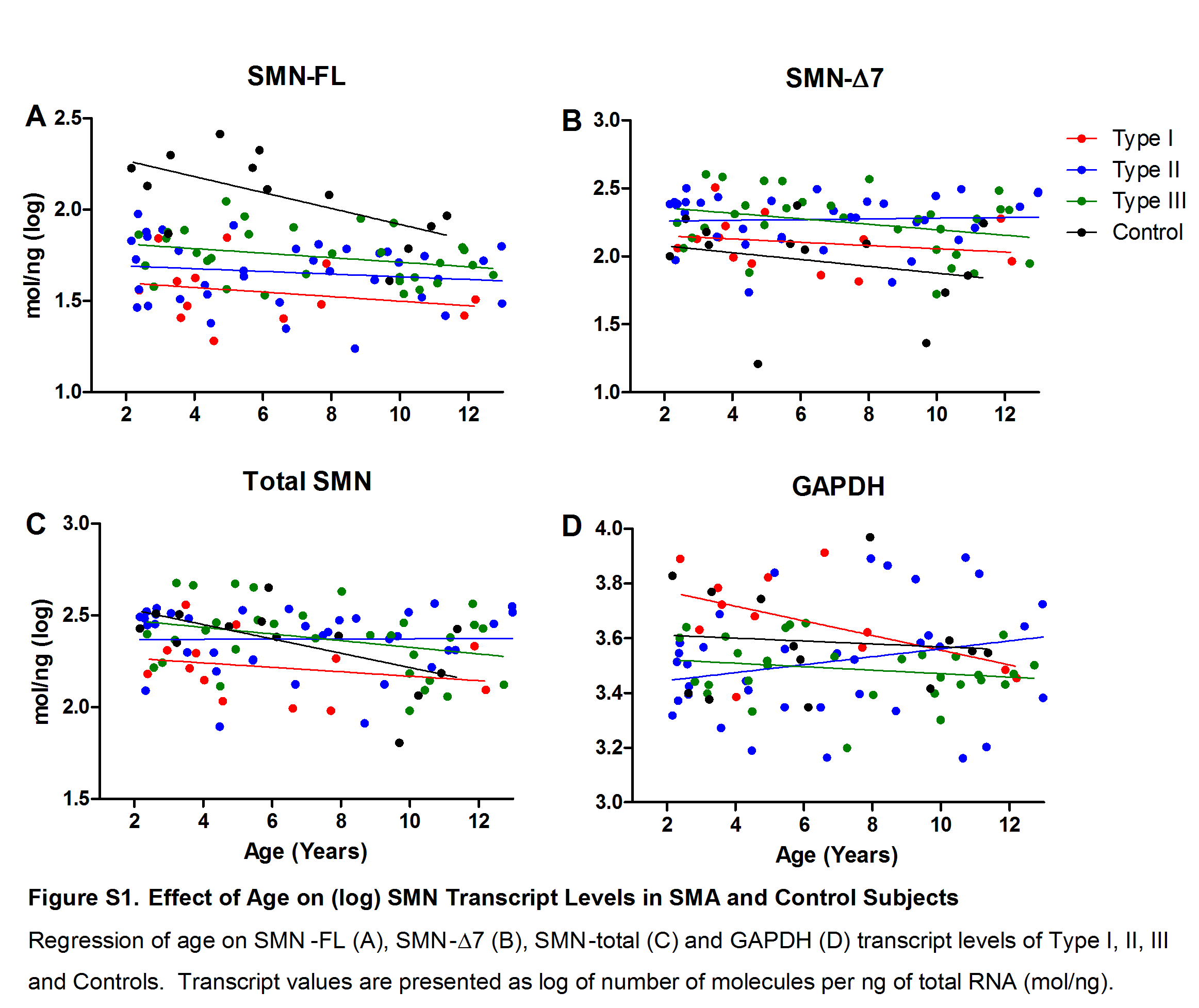

Supplement: Figure S1 — Effect of Age on (log) SMN Transcript Levels in SMA and Control Subjects. Regression of age on SMN-FL (A), SMN-Δ7 (B), SMN-total (C) and GAPDH (D) transcript levels of Type I, II, III and Controls. Transcript values are presented as log of number of molecules per ng of total RNA (mol/ng). (TIF) [file pone.0033572.s001.tif]
