# Supplementary material for: Evaluation of SMN Protein, Transcript, and Copy Number in the Biomarkers for Spinal Muscular Atrophy (BforSMA) Clinical Study
Source: PLoS One. 2012 Apr 27;7(4):e33572. doi: 10.1371/journal.pone.0033572 (PMC3338744; doi:10.1371/journal.pone.0033572)
Supplement: Appendix S1 — BforSMA final NERI IRB-approved protocol. (DOC) [file pone.0033572.s003.doc]

| **Pilot Study of Biomarkers** **for Spinal Muscular Atrophy**  **(BforSMA)** |
| --- |
| | Issue Date: May 30, 2008 | | --- | | Sponsor:  Spinal Muscular Atrophy Foundation  888 Seventh Avenue, Suite 400  New York, NY 10019  Coordinating Center:  New England Research Institutes  9 Galen Street  Watertown, MA 02472 | |
| This protocol is provided to you as a principal investigator, potential investigator or consultant for review by you, your staff and Institutional Review Board/Ethics Committee. The information contained in this document is privileged and confidential and, except to the extent necessary to obtain informed consent, must not be disclosed unless such disclosure is required by federal or state law or regulations. Persons to whom the information is disclosed in confidence must be informed that the information is confidential and must not be disclosed by them. |

CONTACT LIST

| Coordinating Center:  Address:  Phone:  Contacts:  Fax:  E-Mail: | New England Research Institutes (NERI)  9 Galen Street  Watertown, Massachusetts 02472  617-923-7747  Margaret Bell, Program Manager x 522  Carol Assang, Senior Clinical Research Associate x 285  Olga Novikova, Data Manager x455  Rebecca Li, x 243  617-673-9547  SMAProject@neriscience.com |
| --- | --- |
| Study Sponsor:  Address:  Phone: | Spinal Muscular Atrophy Foundation  888 Seventh Avenue, Suite 400  New York, New York 10019  646-253-7100 |
| Medical Monitor:  Address:  Phone:  Fax:  Pager:  E-Mail: | Mustafa Sahin, MD, PhD  Enders 461, Department of Neurology  Children’s Hospital Boston  300 Longwood Avenue  Boston, MA 02115  617-355-2711 (office) |

**TABLE OF CONTENTS**

**ACRONYMS and ABBREVIATIONS 4**

**SYNOPSIS 5**

INTRODUCTION [8](#__RefHeading___Toc200424880)

1.1 Background Information [8](#__RefHeading___Toc200424881)

1.2 Study Rationale [9](#__RefHeading___Toc200424882)

2 OBJECTIVES [10](#__RefHeading___Toc200424883)

2.1 Primary Objective [10](#__RefHeading___Toc200424884)

2.2 Secondary Objectives [10](#__RefHeading___Toc200424885)

3 STUDY DESIGN [10](#__RefHeading___Toc200424886)

4 STUDY POPULATION [10](#__RefHeading___Toc200424887)

4.1 Inclusion Criteria [11](#__RefHeading___Toc200424888)

4.2 Exclusion Criteria [11](#__RefHeading___Toc200424889)

5 Trial Enrollment [12](#__RefHeading___Toc200424890)

5.1 Screening Procedures [12](#__RefHeading___Toc200424891)

5.2 Subject Enrollment and Site Enrollment caps [12](#__RefHeading___Toc200424892)

5.3 Replacement of Subjects [12](#__RefHeading___Toc200424893)

6 STUDY PROCEDURES [13](#__RefHeading___Toc200424894)

6.1 Informed Consent / Informed Assent [13](#__RefHeading___Toc200424895)

6.2 Schedule of Study Measurements [13](#__RefHeading___Toc200424896)

6.3 Screening Procedures [13](#__RefHeading___Toc200424897)

6.4 Study Procedures [13](#__RefHeading___Toc200424898)

7 STUDY MEasurements [14](#__RefHeading___Toc200424900)

7.1 Motor Function Assessment [14](#__RefHeading___Toc200424901)

7.2 10-meter Timed Walk Test [14](#__RefHeading___Toc200424902)

7.3 Pulmonary Function [14](#__RefHeading___Toc200424903)

7.4 Triceps Skin Fold Thickness [14](#__RefHeading___Toc200424904)

7.5 Height, Weight and Ulnar Length [15](#__RefHeading___Toc200424905)

7.6 BMI Z Score [15](#__RefHeading___Toc200424906)

7.7 Specimen Collection [15](#__RefHeading___Toc200424907)

8 SAFETY MONITORING [15](#__RefHeading___Toc200424908)

8.1 Adverse Event, Serious Adverse Event, and Unanticipated problems Definitions [15](#__RefHeading___Toc200424909)

8.1.1 Adverse Event (AE) [15](#__RefHeading___Toc200424910)

8.2 Unanticipated Problems [15](#__RefHeading___Toc200424911)

8.3 Classification of Adverse Events Documentation [16](#__RefHeading___Toc200424912)

8.4 Adverse Event Intensity [16](#__RefHeading___Toc200424913)

8.5 Serious Adverse Event Intensity [16](#__RefHeading___Toc200424914)

8.6 Adverse Event Expectedness [16](#__RefHeading___Toc200424915)

8.7 Adverse Event Causality [17](#__RefHeading___Toc200424916)

8.8 Adverse Event Reporting [17](#__RefHeading___Toc200424917)

8.9 Unanticipated Problems [17](#__RefHeading___Toc200424918)

8.10 Adverse Events [17](#__RefHeading___Toc200424919)

8.11 Serious Adverse Events: [17](#__RefHeading___Toc200424920)

9 TERMINATION OF STUDY PARTICIPATION [18](#__RefHeading___Toc200424921)

10 TRAINING AND DATA MANAGEMENT [18](#__RefHeading___Toc200424922)

10.1 Training Description [18](#__RefHeading___Toc200424923)

10.2 Data Management and Quality Control [18](#__RefHeading___Toc200424924)

10.3 Data Entry and Editing [19](#__RefHeading___Toc200424925)

10.4 Case Report Form Completion [20](#__RefHeading___Toc200424926)

10.5 Specimen Collection Management [20](#__RefHeading___Toc200424927)

10.6 Training on Specimen Collection: [20](#__RefHeading___Toc200424928)

10.7 Data Security and Integrity [20](#__RefHeading___Toc200424929)

11 STATISTICAL CONSIDERATIONS [21](#__RefHeading___Toc200424930)

11.1 Power Calculations [21](#__RefHeading___Toc200424931)

11.2 Statistical Analysis [21](#__RefHeading___Toc200424932)

11.3 Interim Analysis [22](#__RefHeading___Toc200424933)

11.4 Potential Risks and Benefits [22](#__RefHeading___Toc200424934)

11.5 Limitations of the Study [22](#__RefHeading___Toc200424935)

12 PROTOCOL ADMINISTRATION [23](#__RefHeading___Toc200424936)

12.1 Good Clinical Practices [23](#__RefHeading___Toc200424937)

12.2 Ethical Considerations [23](#__RefHeading___Toc200424938)

12.3 Protocol Compliance [23](#__RefHeading___Toc200424939)

12.4 Subject Confidentiality [24](#__RefHeading___Toc200424940)

12.5 Specimen Retention [24](#__RefHeading___Toc200424941)

12.6 Record Retention [24](#__RefHeading___Toc200424942)

12.7 Study Termination by the Sponsor [24](#__RefHeading___Toc200424943)

12.8 Publication and Use of Information [25](#__RefHeading___Toc200424944)

13 REFERENCES [25](#__RefHeading___Toc200424945)

**ACRONYMS and ABBREVIATIONS**

AE Adverse event

CRF Case report form

FDA Food and Drug Administration

FVC Forced vital capacity

GCP Good clinical practice

ICH International Conference on Harmonization

IRB/IEC Institutional Review Board/Independent Ethics Committee

MHFMS Modified Hammersmith Functional Motor Scale

NERI New England Research Institutes

OHRP Office for Human Research Protections

SAE Serious adverse event

SMA Spinal Muscular Atrophy

SMN Survival motor neuron (gene or protein)

| **Pilot Study of Biomarkers** **for Spinal Muscular Atrophy**  **(BforSMA)** |
| --- |

**SYNOPSIS**

**RATIONALE**

Spinal Muscular Atrophy (SMA) is one of the two most common inherited children’s neuromuscular disorders. There currently is no cure and there are no therapeutics approved to slow progression of the disease. SMA is characterized by a loss of alpha motor neurons in the spinal cord, severe atrophy of proximal muscles and progressive debility and disability due to respiratory, gastrointestinal and functional complications of the disease.

Though SMA is a relatively common orphan disease, recruitment of patients for the number of candidate therapies is expected to become rate-limiting for the development of therapeutics. A biomarker is a characteristic that is objectively measured and evaluated, and is an indicator of a pathologic process. The goal in biomarker identification is an empiric interim readout that is directly related to clinical endpoints, i.e., a surrogate endpoint for disease progression and/or treatment effect. The potential advantages of a quality biomarker of disease severity or a network of biomarkers are manifold:

- May replace a distal endpoint with a proximal endpoint, potentially shortening the development time of new therapeutic modalities.
- May be measured more frequently and easily.
- May have greater precision.
- May increase the measured dynamic range of a disease process or treatment effect compared to clinical metrics.
- May result in reduced sample size requirements for clinical studies.
- May lead to expedited decisions concerning the validity of therapeutic interventions.

Thus, the development of biomarkers will have important implications in therapeutics development timelines and more efficiently allocate patient resources into studies with the greatest probability of success.

The goal of this pilot study is to identify a marker or panel of markers in the blood or urine from a wide range of SMA patients that segregates with measures of clinical severity. From this identification of candidate biomarkers, it is hoped that further investigations, both longitudinal natural history and clinical efficacy studies, will verify a biomarker with the sensitivity and specificity that will allow its eventual use as a validated pharmacodynamic marker or surrogate endpoint. In addition, this effort may elucidate biological pathways that may be potential therapeutic targets.

**STUDY DESIGN**

This study will be a multi-center, pilot study enrolling 120 subjects, age 2 to 12 years. The study will enroll subjects from approximately 15 academic pediatric neuromuscular clinics.

Three groups of SMA patients and one cohort of control children will be enrolled according to the classifications below:

- Children with type I SMA (n=15).
- Children with type II SMA (n=45).
- Children with type III SMA (n=40).
- Control children (n=20) will match the gender and age distribution expected in the SMA cohorts. Control children may be either confirmed non-carrier, genetically- related siblings of SMA children or unrelated children.
  - n=5 Females age 2-5.
  - n=5 Females age 6-12.
  - n=5 Males age 2-5.
  - n=5 Males age 6-12.

Each subject will be seen for a single visit, during which an assessment of functional ability, pulmonary status and nutritional status will be performed. In addition, blood and urine specimens will be collected for biomarker assessment and DNA specimens collected for SMN2 copy number. SMN1 genotyping will be conducted for unrelated control subjects only. No therapeutic intervention will occur.

**STUDY OBJECTIVES**

**Primary:**

- To identify candidate blood and urine biochemical markers that correlate with disease severity as determined by the Modified Hammersmith Functional Motor Scale across a range of type I, type II and type III children with Spinal Muscular Atrophy (SMA) (1).

**Secondary:**

- To determine if there are biomarkers from types I-III SMA patients that correlate with SMA type, age at disease onset, 10-meter Timed Walk Test (ambulatory subjects only), pulmonary function, nutritional assessment, SMN protein level, SMN transcript level or SMN2 copy number.
- To determine if identified candidate biomarkers are associated with the disease state through comparison of SMA specimens with control volunteer specimens.
- To determine if there are potential biochemical pathways that may represent targets for therapeutic intervention in SMA.

**KEY INCLUSION CRITERIA**

For all groups:

- Age 2 to 12 years, inclusive.
- In good health in the judgment of the clinical investigator at the time of assessment.

Specific group requirements – SMA children:

- Documented homozygous deletion of SMN1 (exon 7).
- SMA type I defined as the inability ever to sit unaided in the judgment of the clinical investigator.
- SMA type II defined as the ability to ever sit unaided for >30 seconds on a flat surface in the judgment of the clinical investigator.
- SMA type III defined by the ability to ever stand unaided for 30 seconds and walk unaided for >30 ft.

Specific group requirements – Control children:

- Otherwise healthy children that may be either genetically-related siblings of SMA children (confirmed non-carriers) or unrelated children.

**KEY EXCLUSION CRITERIA**

For all groups:

- Systemic or specific-organ illness, including renal, hepatic, cardiac, pulmonary, significant gastrointestinal illness, hematologic or rheumatic disorders requiring ongoing treatment or chronic medication use.
- Any known genetic condition other than Spinal Muscular Atrophy requiring pharmaceutical treatment.
- Use of any putative SMN-enhancing medications or treatments for 14 days prior to enrollment including, valproic acid, phenylbuterate, and hydroxyurea.
- Use of carnitine, creatine, oral albuterol and riluzole for 14 days prior to enrollment.
- Use of any oral prescription medications for 14 days prior to enrollment with the exception of the following medications, which are allowed: anti-reflux medications (e.g. rantidine), constipation or stool softening medications (e.g. polyethylene glycol 3350), stool bulking agents, and inhaled bronchodilator medications and nebulizers (e.g. albuterol).
- Any illness requiring treatment with antibiotics or anti-inflammatory medication within the past 14 days.
- Participation in a clinical trial (except observational studies) within the previous 14 days.

**STUDY EVALUATIONS**

At time of enrollment, the Principal Investigator or designee will obtain detailed clinical history including age of disease onset, diagnosis, motor milestones, loss of motor function (sitting, ambulation). A standardized general physical examination will be performed including vital signs, inspection and evaluation of nutritional balance, pulmonary function and neurological examination.

Nutritional assessment by site P.I. or designee:

- Height, weight, triceps skin fold thickness, and ulnar length.
- Nutritional status question.

Motor function assessment:

- Modified Hammersmith Functional Motor Scale.
- 10-meter Timed Walk Test (ambulatory subjects only).

Pulmonary assessment

- Standardized question about level of respiratory support.
- Forced Vital Capacity (FVC) when > 5 years.

Specimen acquisition

- Blood collected for mRNA, proteomic and metabolomic analysis.
- DNA to determine SMN2 copy number.
- DNA to determine SMN1 genotyping (non-genetically related controls only).
- Non-first morning void urine for proteomic and metabolomic analysis.

**RATIONALE FOR NUMBER OF SUBJECTS**

- The number of subjects was selected based on clinical and statistical considerations.
- The sample size for the SMA subjects was selected to achieve 83% power for the primary outcome of Modified Hammersmith Functional Motor Scale, using an elastic net regression analysis, assuming a 0.75 correlation between the observed and theoretical outcomes. The power estimate is based on an average of 100 simulated datasets using data available from the original Hammersmith Scale.
- The sample size of 20 control subjects was selected to achieve at least 90% power to detect a univariate biomarker with a mean-fold change (MFC) of 1.5 when the false discovery rate is controlled at 0.05.
- In both power calculations it was assumed that 10% of profiled analytes are true biomarkers, and that variance of analytes is equal to 0.2.

# INTRODUCTION

- 1. Background Information

Spinal muscular atrophy (SMA) is a common and severe autosomal recessive disease, affecting all races with an overall incidence in the United States that rivals cystic fibrosis and sickle cell disease (2).

SMA manifests across a continuous spectrum of severity and age of disease onset that has been divided into four groups: type I (severe infantile acute SMA, or Werdnig-Hoffmann disease); type II, (infantile chronic SMA); type III (juvenile SMA, or Wohlfart-Kugelberg-Welander disease); and type IV (adult-onset SMA). The three most severe forms manifest in children, and are divided by maximal motor function achieved: type I infants never sit, type II sit, but never walk, and type III are able to walk at least for a short time (3). All types are caused by homozygous protein-null mutations (deletion, conversion, or missense) of the SMN1 gene (4).

Across its range of manifestation, the common clinical feature of SMA is diffuse, symmetric weakness and atrophy of skeletal muscle due to denervation. This is caused by degeneration of the anterior horn cells (motor neurons) of the spinal cord and brainstem. The understanding of SMA has expanded greatly since the discovery of the genetic basis. In humans a large region of chromosome 5q is duplicated, with the result that two copies of SMN reside on each chromosome. In addition, there is considerable meiotic instability of the SMN-critical 5q duplicated chromosomal region owing to differences in number, copy number, and orientation of multiple markers (5). The two copies of SMN – designated SMN1 and SMN2 – differ in only one base pair, a synonymous protein-coding change in exon 7 (6). Most SMN protein is derived from the SMN1 gene, but the SMN2 gene contributes a small amount of normal SMN protein due to a leaky splice site at the intron 6-exon 7 boundary. If SMN1 is lost on both alleles, the protein translated from SMN2 is sufficient to rescue embryonic lethality, but insufficient to prevent manifestation of the SMA syndrome. The range of clinical severity observed in SMA varies in part as a consequence of variation in copy number of SMN2: greater copy number is associated with a more mild phenotype (7).

SMN is expressed in all tissues and cell types; the relative specificity of the pathogenic process for lower motor neurons is not currently understood. Αlpha motor neurons may have unique susceptibility to a loss in snRNA assembly or SMN may have accessory functions in this cell or its connections (8) (9) (10). SMN protein is found in axons and growth cones (11) (12), and may function to transport mRNAs from the nucleus to synapse. In other models it is localized post-synaptically at the neuromuscular junction and within the Z-bands of striated muscle (13). Therefore the primary pathophysiology of SMA may stem not only from functions within the cell body of the lower motor neuron, but also within the axon, neuromuscular junction or even muscle.

Technology now allows non-assumptive data mining of the human nucleic acid (genome and transcriptome), polypeptide (proteome) and small molecule (metabolome) repertoire. A functional consequence is that this technology can be used to generate a disease ‘signature’ of biochemical markers which segregates with disease process or severity. Because these biochemical changes are assumed to be a consequence of the single gene perturbation in SMA, presumptive markers may mirror the disease pathology. This has important consequences in therapeutics discovery for SMA:

1. It may lead to the generation of sensitive disease biomarkers that are functional surrogates for disease progression and/or therapeutic benefit.
2. It may lead to identification of previously unidentified targets or pathways critical in the manifestation of the disease.
3. It may support hypotheses of pathogenesis that confirm and/or extend our knowledge of the disease process.
   1. Study Rationale

Clinical testing of new therapeutics in SMA requires effective tools for recruiting patients and measuring outcomes pertinent for registration. Because of many variables including age of disease onset and a broad spectrum of severity, no one clinical outcome measure currently represents the gold standard for clinical testing (14). We propose that there may be a biochemical surrogate of disease that may represent a more sensitive and uniformly applicable variable to measure as an interim readout or surrogate endpoint. This pilot protocol is intended to preliminarily identify a panel of biochemical markers that segregate with disease severity as judged by several diagnostic and outcome metrics.

A biomarker is a characteristic that is objectively measured and evaluated, and is an indicator of a pathologic process. The goal in biomarker identification is an empiric interim readout that is directly related to clinical endpoints, i.e., a surrogate endpoint for disease progression and/or treatment effect. The potential advantages of a quality biomarker of disease severity or a network of biomarkers are manifold:

1. May replace a distal endpoint with a proximal endpoint, potentially shortening the development time of new therapeutic modalities.
2. May be measured more frequently and easily.
3. May have greater precision.
4. May increase the measured dynamic range of a disease process or treatment effect compared to clinical metrics.
5. May result in reduced sample size requirements for clinical studies.
6. May lead to expedited decisions concerning the validity of therapeutic interventions.

Thus, the development of biomarkers will have important implications in therapeutics development timelines and more efficiently allocate patient resources into studies with the greatest probability of success.

The goal of this pilot study is to identify a marker or panel of markers in the blood or urine from a wide range of SMA patients that segregates with measures of clinical severity. From this identification of candidate biomarkers, it is hoped that further investigations, both longitudinal natural history and clinical efficacy studies, will verify a biomarker with the sensitivity and specificity that will allow its eventual use as a validated pharmacodynamic marker or surrogate endpoint. In addition, this effort may elucidate biological pathways that may be potential therapeutic targets.

# OBJECTIVES

- 1. Primary Objective
- To identify candidate blood and urine biochemical markers that correlate with disease severity as determined by the Modified Hammersmith Functional Motor Scale across a range of type I, type II and type III children with spinal muscular atrophy (SMA).
  1. Secondary Objectives
- To determine if there are biochemical markers from types I-III SMA patients that correlate with SMA type, age at disease onset, 10-meter Timed Walk Test (ambulatory subjects only), pulmonary function, nutritional assessment, SMN protein level, SMN transcript level, or SMN2 copy number.
- To determine if identified candidate biomarkers are associated with the disease state through comparison of SMA specimens with control volunteer specimens.
- To determine if there are potential biochemical pathways that may represent targets for therapeutic intervention in SMA.

# STUDY DESIGN

This study will be a multi-center, pilot study enrolling 120 subjects, age 2 to 12 years from approximately 15 academic pediatric neuromuscular clinics. Primary care pediatric practices within these institutions may refer patients for the controls. Participating clinic sites will have the laboratory support to process the blood and urine samples appropriately for shipment to central laboratory and biomarker analysis.

Each subject will be seen for a single visit, during which an assessment of functional ability, pulmonary status and nutritional status will be performed. In addition, blood and urine specimens will be collected for biomarker assessment and DNA specimens collected for SMN2 copy number. SMN1 genotyping will only be conducted on control subjects who are genetically unrelated to SMA children. Control subjects have the option to decline the DNA (SMN1 gene analysis) portion of the study. No therapeutic intervention will occur.

# STUDY POPULATION

Three groups of SMA patients and one cohort of control children will be enrolled according to the classifications below:

- Children with type I SMA (n=15).
- Children with type II SMA (n=45).
- Children with type III SMA (n=40).
- Control subjects (n=20) will match the gender and age distribution expected in the SMA cohorts. Control children may be either confirmed non-carrier, genetically- related siblings of SMA children or unrelated children (limit 1 child per family for enrollment in the control cohort).
- n=5 Females age 2-5.
- n=5 Females age 6-12.
- n=5 Males age 2-5.
- n=5 Males age 6-12.
- SMA subjects will be enrolled from approximately 15 academic pediatric neuromuscular clinics.
- Control subjects will be enrolled from pediatric neuromuscular clinics as well as referring pediatric practices within the same institutions.
  1. Inclusion Criteria

For all groups:

1. Age 2 to 12 years, inclusive.
2. In good health (other than SMA) in the judgment of the clinical investigator at the time of assessment.

SMA group requirements:

1. Documented homozygous deletion of SMN1 (exon 7). Genotyping results must be performed by a CLIA certified laboratory and documented via chart review.
2. SMA type I defined as the inability ever to sit unaided in the judgment of the clinical investigator.
3. SMA type II defined as the ability to ever sit unaided for >30 seconds on a flat surface in the judgment of the clinical investigator.
4. SMA type III defined by the ability to ever stand unaided for 30 seconds and walk unaided for >30 ft in the judgment of the clinical investigator.

Control group requirements:

1. Otherwise healthy children that may be either genetically-related siblings of SMA children (genetically confirmed non-carriers), or unrelated children. Only one sibling per family with an SMA subject may be enrolled (includes related and unrelated siblings).
   1. Exclusion Criteria

For all groups:

1. Systemic or specific-organ illness, including renal, hepatic, cardiac, pulmonary, significant gastrointestinal illness, hematologic or rheumatic disorders requiring ongoing treatment or chronic medication use.
2. Any known genetic condition other than Spinal Muscular Atrophy requiring pharmaceutical treatment.
3. Use of any putative SMN-enhancing medications or treatments for 14 days prior to enrollment including, valproic acid, phenylbuterate, and hydroxyurea.
4. Use of carnitine, creatine, oral albuterol or riluzole for 14 days prior to enrollment
5. Use of any oral prescription medications for 14 days prior to enrollment with the exception of the following medications, which are allowed: anti-reflux medications (e.g. rantidine), constipation or stool softening medications (e.g. polyethylene glycol 3350), stool bulking agents, and inhaled bronchodilator medications and nebulizers (e.g. albuterol).
6. Any illness requiring treatment with antibiotics or anti-inflammatory medication within the past 14 days.
7. Any rash (excluding diaper rash or eczema) requiring treatment within the past 7 days.
8. **Any severe asthma attack requiring treatment with oral or parenteral steroids within the past 7 days.**
9. Any fever over 100 degrees Fahrenheit or 38 degrees Celsius within the past 7 days.
10. Any immunization within the past 7 days.
11. Any injury sustained that resulted in a bone fracture or needed stitches within the past 7 days.
12. Any surgery **within the past 7 days.**
13. **Any receipt of anesthesia within the past 7 days.**
14. **Any Emergency Room visit within the past 7 days.**
15. **Any hospitalization within the past 7 days.**
16. Any stomach illness with vomiting within the past 7 days.
17. **Any migraine headache within the past 7 days.**
18. Participation in a clinical trial (except observational studies) within the previous 14 days.

# Trial Enrollment

There is no randomization or blinding protocol for this study as there is no therapeutic intervention.

- 1. Screening Procedures

The Principal Investigator at each clinic site (or designee) and the study coordinator will have responsibility for subject recruitment including a chart review to identify potentially eligible subjects. All screening must comply with the local institution’s Health Insurance Portability and Accountability Act (HIPAA) requirements. Subjects may be screened for eligibility more than once during the accrual period.

- 1. Subject Enrollment and Site Enrollment caps

Subjects will be enrolled based on the definitions listed under inclusion and exclusion criteria. As treatment patterns differ between institutions, the study aims for a broad recruitment over a number of sites rather than 1 or 2 sites dominating the subject pool. This aim is balanced by the logistical concern of not significantly hampering recruitment in a rare disease. Subject caps will be lifted 3 months after the first subject is enrolled in the study. To enable recruitment to proceed smoothly, sites will be able to access real-time recruitment information by cohort via the web through the data management system (ADEPT). The distributions of sex and age of SMA cases will be monitored regularly, and the plan for recruitment of controls of appropriate sex and ages may be altered in order to match distributions.

The following subjects caps (maximum number of subjects allowed per site) for each cohort are defined below:

Table 1: Subject Caps Per Site

|  | Total subjects | Subject Cap per site |
| --- | --- | --- |
| Type I | 15 | 5 |
| Type II | 45 | 10 |
| Type III | 40 | 10 |
| Controls | 20 | 7 |
| TOTAL | 120 |  |

- 1. Replacement of Subjects

Subjects may be replaced by additional subjects in the same cohort prior to the close of the enrollment period if:

- Subject’s specimen(s) collected are lost in transit.
- Subject’s specimen(s) are broken upon receipt.
- Subject’s specimen(s) are compromised or unanalyzable (i.e. hemolyzed) upon receipt.
- Specimens of a particular type do not meet minimum volumes for processing.
- No specimens of a particular type are collected during the visit.
- Study assessments required for the primary or secondary outcome are not collected.

Any clinical data collected or usable specimens for the subjects replaced may be analyzed and used in the statistical analysis.

# STUDY PROCEDURES

- 1. Informed Consent / Informed Assent

Signed and dated IRB-approved informed consent must be obtained from the subject’s parent or guardian before any study-specific screening procedures are performed. The Principal Investigator or approved designee will obtain informed consent from the parent or guardian and an informed assent from the pediatric subject (according to local institutional IRB guidelines) after full review of the pros and cons of the study. The possible benefits and complications of participation will be explained in detail. Every effort will be made to ensure that the subject and parent/guardian fully comprehend the nature of the study and the details of his or her participation. A copy of the consent form will be provided to the parent/guardian and subject. The informed consent process must be documented in the subject’s medical chart.

- 1. Schedule of Study Measurements

Subjects will be screened for eligibility according to the inclusion/exclusion criteria and then will be enrolled and have additional study evaluations on the same day. The purpose of the screening is to identify eligible subjects and to eliminate evidently unsuitable subjects before the enrollment process is started. In some instances, at the site’s discretion, the screening may occur on a separate day than study procedures (for example to confirm carrier status of siblings of SMA subjects). It is anticipated that the duration for all the screening and study procedures will be between 2-4 hours.

- 1. Screening Procedures

Each clinical site will maintain a Screening Log of all subjects screened for the trial. This log will record the reason for any screen failure. Confidentiality of subject data will be maintained throughout the trial. No patient identifiers will be used and all data entered into the trial database will be under code.

All screening procedures are the same for the SMA subjects and the control group.

1. Medical review of inclusion and exclusion criteria.
2. Temperature.
3. Those subjects deemed eligible by the Principal Investigator or designee will complete additional evaluations for Enrollment.
   1. Study Procedures

All study procedures are the same for the SMA subjects and the control group with the exception of collection of DNA for SMN1 genotyping for control subjects only (refer to 6c below).

1. Medical history including motor milestones, age of disease onset for children with SMA, loss of motor function (sitting, ambulation) for children with SMA.
2. Physical Examination including Neurological assessment ,vital signs: sitting BP, pulse and respirations and height, weight and ulnar length.
3. Nutritional assessment by Principal Investigator or designee:
4. Triceps skin fold thickness.
5. Nutritional status question about level of solid food intake.
6. Motor function assessment
7. Modified Hammersmith Functional Motor Scale.
8. 10-meter Timed Walk Test (ambulatory subjects only).
9. Pulmonary assessment
10. Standardized question about level of respiratory support.
11. Forced Vital Capacity (FVC) when subject is greater or equal to 5 years.
12. Specimen acquisition
13. Blood collected for mRNA, proteomic and metabolomic analysis.
14. DNA to determine SMN2 copy number.
15. DNA to determine SMN1 genotyping (genetically unrelated controls only). Subjects have the option to decline this test.
16. Blood collected for SMN protein level.
17. Non-first morning void urine for proteomic and metabolomic analysis.

# STUDY MEasurements

- 1. Motor Function Assessment

The Modified Hammersmith Functional Motor Scale (15) provides a tool for evaluation of motor function in pediatric subjects with SMA type II and type III. The Modified Hammersmith Functional Motor Scale will be performed according to the Study Manual of Operations. A score of “0” is acceptable for those unable to perform any of the measurements (e.g., type I SMA subjects). In this study, both control subjects and SMA subjects will undergo the assessment as the scale is being used as the primary outcome. A minimal amount of equipment is required to perform the evaluation and the total duration is ~20 to 30 minutes to complete. Each site will have a clinically trained person performing these evaluations. It is anticipated that for control subjects, the assessment will require a minimum amount of effort and will take approximately 10 minutes to complete.

- 1. 10-meter Timed Walk Test

A 10-meter Timed Walk Test will be performed on all ambulatory SMA subjects and controls according to the Study Manual of Operations. This test compares higher functioning subjects with SMA and control subjects in their ability to walk 10 meters. (16, 17, 18)

- 1. Pulmonary Function

On subjects age >5 years, spirometry will be performed according to the Manual of Operations to measure the maximal volume of air forcibly exhaled from the point of maximal inhalation (forced vital capacity, FVC). The test will be performed 3 times in a sitting position. Subjects unable to sit may perform the FVC in a supine position and document on the appropriate CRF. Subjects who are unable to perform or complete the FVC tests may still participate in the study.

- 1. Triceps Skin Fold Thickness

The measurement of skin fold thickness of the subject’s upper arm will give an estimate of body composition and will be correlated with BMI Z score. Variability of measurement will be decreased by detailed standard procedures in the Manual of Operations and hands-on training.

- 1. Height, Weight and Ulnar Length

To calculate the BMI Z score (refer to 7.5) a subject’s height and weight is measured. Height will be measured to the nearest centimeter or tenth of an inch; weight will be measured to the nearest tenth of a pound or nearest gram. The subject is to wear light clothing with socks and shoes removed. Ulnar length will be measured because height measurement may be unreliable when neuromuscular weakness or spinal deformity is present (19). Ulnar length will be measured to the nearest centimeter or tenth of an inch as the distance between the proximal end of the ulna and the tip of the styled process. Detailed standard procedures for all these measurements are in the Manual of Operations.

- 1. BMI Z Score

The BMI Z Scale (20) is used to determine a child’s body mass index (BMI). The BMI-percentile-for-age calculator automatically adjusts for differences in height, age and gender, making it is one of the best tools for evaluating a growing child's weight. Many SMA children have a higher average BMI related to their lack of motor activity. The BMI Z Score will be automatically calculated in the Data Management System (ADEPT) upon data entering the height, weight, and ulnar length.

- 1. Specimen Collection

Up to approximately 10-mL sample of blood will be collected for gene expression (mRNA), proteomic and metabolomic analysis. All samples will be shipped and stored at the central laboratory until the end of the study where they will be shipped to the designated genomic laboratory (DNA and mRNA samples) and biomarker laboratory (plasma) for analysis. From the blood, DNA will be extracted to determine SMN2 copy number and SMN1 genotype (for genetically unrelated controls only). Please refer to the Manual of Operations for detailed processing and shipping instructions.

Up to 10-mL sample of urine will be collected for proteomic and metabolomic analysis. All samples will be shipped and stored at the central laboratory until the end of the study where they will be shipped to the designated biomarker laboratory for analysis. Please refer to the Manual of Operations for detailed processing and shipping instructions.

# SAFETY MONITORING

This study is not an intervention study. However, a major component of safety monitoring is ascertainment and reporting of adverse events, including adverse reactions to study procedures. The approach to these activities for this trial is summarized in the sections that follow. Adverse events will be defined by standard nomenclature as defined below.

- 1. Adverse Event, Serious Adverse Event, and Unanticipated problems Definitions
     1. Adverse Event (AE)

An adverse event (AE) is any untoward medical occurrence in a subject, including any abnormal sign, symptom, laboratory abnormality or disease, temporally associated with the subject’s participation in the research, whether or not considered related to the subject’s participation in the research. (21)

- 1. Unanticipated Problems

OHRP considers unanticipated problems, in general, to include any incident, experience, or outcome that meets all of the following criteria:

- Unexpected (in terms of nature, severity, or frequency) given the study procedures that are described in the protocol related documents, such as the IRB approved research protocol and informed consent and assent documents; and the characteristics of the subject population being studied;
- Related or possibly related to participation in the research (possibly related means there is a reasonable possibility that the incident, experience, or outcome may have been caused by the procedures involved in the research; and
- Suggests that the study places subjects or others at a greater risk of harm (including physical, psychological, economic, or social harm) than was previously known or recognized.

If the answer to all three questions is yes, then the event is an unanticipated problem and must be reported to appropriate entities (22). To assess these three questions requires that events be classified as to seriousness, expectedness, and potential relationship to the study participation.

- 1. Classification of Adverse Events Documentation

Monitoring adverse events requires that they be classified as to intensity, expectedness, and potential relationship to the study procedures. All adverse events occurring within 24 hours of the signing of the informed consent, regardless of intensity, expectedness or causal relationship, will be recorded.

- 1. Adverse Event Intensity

Intensity of an adverse event will be defined as follows:

Mild (1): Awareness of event, but easily tolerated

Moderate (2): Discomfort enough to cause some interference with daily activities

Severe (3): Inability to perform normal daily activities

- 1. Serious Adverse Event Intensity

A serious adverse event is any untoward medical occurrence that:

1. Results in death.
2. Is life threatening (refers to an event in which the subject was at risk of death at the time of the event; it does not refer to an event which hypothetically might have caused death if it were more severe).
3. Requires hospitalization or prolongation of existing hospitalization.
4. Results in persistent or significant disability/incapacity or
5. Results in a congenital anomaly/ birth defect.
6. A medical event that may not be immediately life-threatening or result in death or hospitalization but that may jeopardize the subject or may require intervention to prevent a serious outcome, may also be considered serious (e.g. intensive treatment in an emergency room or at home for allergic bronchospasm; blood dyscrasias or convulsions that did not result in hospitalization; or development of drug dependency or abuse). Medical and scientific judgment must be exercised when classifying events as serious.
   1. Adverse Event Expectedness

All adverse events will be evaluated as to whether their occurrence was expected.

**Unexpected**: An unexpected adverse event or adverse reaction to study procedures is one for which the nature or severity is not consistent with either:

1. Known information associated with the procedures and described in the protocol, consent/assent form, and other relevant sources of information; or
2. The expected natural progression of any underlying disease, disorder, or condition of the subject(s) experiencing the adverse event and the subject’s predisposing risk factor profile for the adverse event.

**Expected**: An event is considered expected if is known to be associated with the study procedures or the expected natural progression of the subjects’ underlying disease or condition. For this protocol, expected events include the following.

1. In the subjects with SMA:
   1. Fatigue
   2. Respiratory change or respiratory event
2. In subjects of this age range:
   1. Bruising at phlebotomy site
   2. Adverse Event Causality

All adverse events will be assessed as to determine causality

1. Not related: The event is clearly related to other factors, such as the subject’s clinical state, or non-study procedure.
2. Possibly related: The event follows a compatible temporal sequence form the time of the study procedure, but could have been produced by other factors such as the subject’s clinical state or non-study procedure.
3. Probably related: The event follows a reasonable temporal sequence form the time of the study procedure, and cannot be reasonably explained by other factors such as the subject’s clinical state or non-study procedure.
   1. Adverse Event Reporting
   2. Unanticipated Problems

In monitoring adverse events, it is essential to determine whether an adverse event represents an unanticipated problem (i.e., meets all 3 criteria specified in Section 8.1) that needs to be reported to appropriate entities based on OHRP guidelines (23). The institution at which the subject experienced an adverse event determined to be an unanticipated problem must report to the OHRP.

- 1. Adverse Events

All adverse events will be captured for a 24 hour period starting from the time the subject signs informed consent. All events will be reported within 7 days of notification of the event. All serious adverse events will be assessed by a qualified medical monitor appointed by the Coordinating Center.

- 1. Serious Adverse Events:

All serious (i.e. not fatal or not life-threatening) adverse events that are unexpected and considered possibly or probably related to the study procedure will be reported to the Coordinating Center within 7 days of learning of the event. All fatal or life-threatening adverse events are to be reported to the Coordinating Center within 24 hours of learning of the event.

If the serious, unexpected adverse event is associated with study procedures, the Coordinating Center will notify the appropriate regulatory agency (ies) and all participating investigators on an expedited basis. It is the responsibility of the investigator to promptly notify the Institutional Review Board/Independent Ethics Committee (IRB/IEC) of all adverse events according to local IRB/IEC policies.

# TERMINATION OF STUDY PARTICIPATION

Subjects may be withdrawn from the study for the following reasons:

- Subject (or legal guardian) declines further study participation.
- In the investigator’s judgment, it is in the subject’s best interest.

# TRAINING AND DATA MANAGEMENT

- 1. Training Description

The Coordinating Center will provide a central protocol training session for all the PIs and study coordinators (and OTs or PTs if they will be performing the Modified Hammersmith Functional Motor Scale assessment). The training session will include using real subjects or video on anthropometric measurements and the Modified Hammersmith Functional Motor Scale assessment, 10-meter Timed Walk Test (ambulatory subjects only) and spirometry testing to ensure consistency and quality across clinic sites. Hands-on training on the Coordinating Center’s web-based Advance Data Entry and Protocol Tracking (ADEPT) system will also be conducted for all study coordinators. Study coordinators must successfully complete an ADEPT certification package at the end of the training session prior to gaining access in ADEPT to ensure database proficiency. The Coordinating Center will conduct on-site in-person training sessions for PIs and study coordinators who are unable to attend the central training session. As accuracy of the specimen handling is essential to achieve study goals, training on proper collection, handling, storage and shipping of blood and urine specimens will be provided during the central training session or at participating sites. All key site personnel who handle and prepare the specimens will be trained by the Central Laboratory and monitored by the Central Laboratory and the Coordinating Center.

- 1. Data Management and Quality Control

A customized data management system will be developed for this study using the Coordinating Center’s Web-based Advanced Data Entry and Protocol Tracking (ADEPT) system. ADEPT is a customizable, secure, web-based system with an Oracle relational database engine, which allows direct data entry from multiple study sites. Information entered into the data entry system will be by subject study identification number; names will not be linked with subject data in the database. Sites will maintain records in secure areas linking the subject name with the identification number assigned for the study. Sites will have full access to their own data and be able to view these data remotely. Study staff will not be able to view subject data associated with other sites. Users associated with the SMA Foundation will have access to cross-site aggregate data. The Coordinating Center’s ADEPT software is designed to be compliant with 21 CFR Part 11 (24).

The data management system for the study will integrate all aspects of study data collection including:

- Participant screening and enrollment data.
- Web-based entry and on-line editing of all study related CRFs.
- Electronic signing of CRFs by site PIs.
- Matching cases and controls.
- Enforcing group balance requirements within sites.
- Collection, processing and tracking of specimens and results to the Central laboratory where specimens will be stored and batched until final distribution to the genomic laboratory and biomarker laboratory (see Specimen Collection Management in protocol section 10.3 and the Manual of Operations).
- Uploading genomic and biomarker results.
- Provide real-time reports on enrollment, data quality, protocol violations, etc. to the sponsor.

Sites enter data via a customized ADEPT secure web application. This system has been specifically designed to support reliable secure data entry for clinical research purposes. The system provides real-time field level validations, context sensitive help and automatic query generation. Data entry forms use browser based logic to enforce proper validations of all data fields and proper skip patterns within study data forms. Interim background data submittals prevent loss of data due to interruption of internet connections.


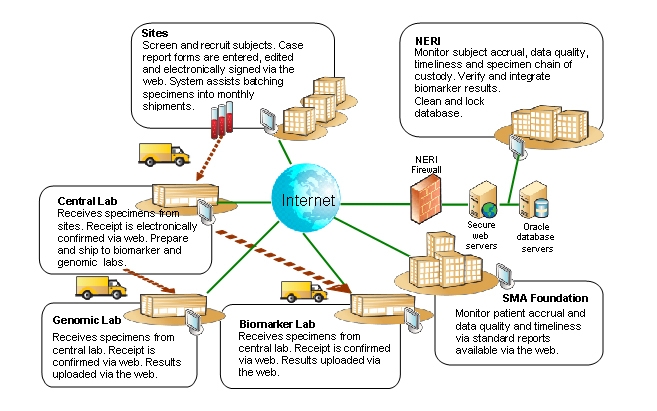


Figure 1. Data Management System

- 1. Data Entry and Editing

The data entry system will include a number of standard features designed to ensure consistently high quality data. Each question on the study form will be associated with a validation, and validations will be executed in real-time during data entry. If a response to a particular question falls outside the range of allowable values specified in the validation for that question, the user will be alerted so that the error can be corrected immediately. Validations will include both inter- and intra-instrument data checks. In addition to alerting the user to invalid entry of items, edit reports will be automatically generated at the completion of data entry for a form. These edit reports will provide the information necessary to investigate any data entry errors or resolve questions regarding out-of-range or questionable values. Edits reports will list subject I.D. number, instrument name, and a detailed description of why each specific data item was flagged. These edits reports can be printed out and reviewed by a supervisor, and returned to the data collector for resolution.

- 1. Case Report Form Completion

CRFs must be completed for each screened and enrolled study subject. It is the investigator’s responsibility to ensure the accuracy, completeness, legibility and timeliness of the data reported in the subject’s CRF. All CRF entries should be derived from, and consistent with, source documentation. Any discrepancies should be explained. Source documentation should indicate the consenting process, the subject’s participation in the study and should document the dates and details of study procedures, adverse events and subject status.

- 1. Specimen Collection Management

Ensuring the accuracy of blood and urine specimens for this trial is paramount. The Coordinating Center and the Central Laboratory have quality control methods in place to ensure this accuracy. Aliquot tubes are linked, and tracked to allow tracing back to the original parent tube. The complete electronic chain of custody has in place will allow the SMA data management system to monitor and report on the critical processes involved in specimen collection, shipping and results. There is immediate feedback between the clinical site, the Coordinating Center and the Central Laboratory when a shipment of specimen samples is sent from the clinical site and the Central Laboratory receives these samples. The final shipment of specimens to the genomic laboratory and biomarker laboratory is also closely monitored for any discrepancies and damage. Reports will be programmed to show by specimen type:

- Count and % specimens compliant with specimen collection.
- Count and % specimens compliant with specimen processing and aliquotting.
- Count and % specimens successfully shipped from sites.
- Count and % specimens received intact by central lab.
- Count and % specimens with successful receipt of corresponding results from biomarker lab.

- 1. Training on Specimen Collection:

ADEPT automatically generates manifests used by sites during the shipping processes. Manifests list the specimens available at the site for shipment. Upon confirmation of shipment, an electronic version of the manifest is forwarded to the central lab, where receipt is also electronically confirmed. For each confirmed specimen at the central laboratory, the data management system will anticipate an appropriate set of results.

Sites will be required to enter the following data into ADEPT:

- Specimen collection date, time, and volume.
- Specimen processing completion date, time, and processing temperature
- Sample storage date, time, and temperature prior to shipment.
- Sample shipment date, time, and temperature.

ADEPT will alert the site if any of the parameters entered are out of range or invalid.

- 1. Data Security and Integrity

The Web-based components of the data management system utilize several levels of security to ensure privacy and integrity of the study data as noted below:

- Web access to ADEPT requires use of assigned user names and passwords.
- Passwords are changed every 90 days.
- Web-based data entry uses secure socket layer (SSL) data encryption.
- Access to any study-specific system features are controlled by Oracle database rights and privileges.
- A full Oracle back up is done on a daily basis and stored offsite.
- Duplicate NT servers are available to replace Oracle or Web Server.
- Primary Identification is via study I.D.
- Access to electronic linkage limited by Oracle Database Administrator.
- Access to hard copies linkage kept in locked cabinets by Clinical Center Coordinators.
- The Coordinating Center firewall limits which internet protocols are allowed to access the Web server.
- No direct access is allowed to the Oracle server from the internet.
- The Coordinating Center’s firewall monitors for unusual (hacker) activity and automatically notifies the Coordinating Center IS staff.

All study data will be stored on the Coordinating Center’s Microsoft NT-based, Oracle server. Access to data on this server (from both inside and outside the data center) is controlled by Oracle’s extensive security features. The Oracle archiving and back-up system ensures minimal data loss, even in the most catastrophic system failure.

# STATISTICAL CONSIDERATIONS

- 1. Power Calculations

A sample size of 15 Type I, 45 Type II, and 40 Type III SMA cases provides 83% power for the primary outcome of Modified Hammersmith Functional Motor Scale using an elastic net multivariate regression analysis of Modified Hammersmith Functional Motor Scale on composite biomarker sets, at level 0.05, assuming a correlation between observed and theoretical outcomes of 0.75. This power calculation also assumes that 10% of analytes are true biomarkers, a variance of 0.2 for all analytes, with 100 analytes profiled. The power estimate is based on an average of 100 simulated datasets using data available from the original Hammersmith Scale.

The power to detect a mean fold change (MFC) of 1.3 in the key secondary outcome of SMA type, under the same assumptions as above, is 89% for univariate analysis of variance (ANOVA), when the false discovery rate (FDR) is controlled at level 0.10. For multivariate PAM analysis (Prediction Analysis of microarrays), power is 83% to detect a multivariate biomarker set with MFC of 1.2, when the theoretic prediction accuracy is 61%. These power calculations assume monotone identical differences between SMA types, and are based on an average of 100 simulated datasets for ANOVA and 1000 for PAM.

Additionally, the sample size of 100 SMA cases and 20 controls, under the same assumptions as above, provides 92% power to detect a univariate biomarker with a MFC of 1.5 between SMA and control cases (t-test) when the FDR is controlled at level 0.05.

- 1. Statistical Analysis

The primary outcome for this study is the Modified Hammersmith Functional Motor Scale. A score of “0” is acceptable for those unable to perform any of the measurements (e.g., type I SMA subjects). Secondary outcomes include SMA type, timing of symptoms (age of disease onset, age of maximum function), motor milestones, nutritional assessment (height, weight, triceps skin fold thickness, ulnar length, nutritional question), pulmonary assessment (standardized question about level of respiratory support, FVC when ≥ 5 years), SMN protein, SMN transcript and SMN2 copy number. Descriptive statistics (e.g., means, standard deviations, percents) of all outcomes will be calculated for SMA and control cases, as well as by SMA type. The populations will also be described by vital signs, loss of motor function, and age appropriate neurological exam results.

In order to find disease-specific biomarkers, comparison of biomarkers between SMA and control cases will be performed using regression analysis, controlling for sex and age. Correlations of biomarkers with outcomes will be conducted within SMA cases, as well as in the combined population of SMA and control cases.

To model the correlation of each individual biomarker with the Modified Hammersmith Functional Motor Scale, univariate regression analyses will be performed, controlling for sex and age. Additionally, elastic net multivariate regression (25) will be used to find a composite set of biomarkers that correlates with outcome.

Univariate regression and elastic net multivariate regression will also be used for continuous secondary outcomes (e.g., age of disease onset, height, weight). For categorical outcomes (e.g., SMA type), analysis of variance (ANOVA), controlling for sex and age, will be performed to discover individual biomarkers that vary by outcome. Likewise, PAM (Prediction Analysis of microarrays) (26) will be used to try to find a composite set of biomarkers that varies by outcome.

In all analyses a p-value ≤ 0.05 will be considered statistically significant. The false discovery rate (27) will also be controlled. If there is an extensive amount of missing data, or data appears not to be missing completely at random, then multiple imputations will be considered. Standard statistical software packages (e.g., SAS) will be used.

- 1. Interim Analysis

No interim analysis for safety or efficacy will be performed during this study.

- 1. Potential Risks and Benefits

Potential risks to study subjects are minimal. Blood will be drawn in volumes that are approximately half (or less than half) of the maximum blood draw allowed for children (by weight) (28) (29). SMN2 copy number will be genotyped from DNA specimens for all cohorts. SMN1 gene analysis will only be done on those control subjects with no genetic relationship to SMA children. SMA subjects will be pre-diagnosed and therefore no further risks are anticipated from a genetic point of view. The control subjects may opt out of the DNA portion (SMN1 gene analysis) of the study. The control subject’s risk is that the study does not benefit the individual directly.

Potential benefits are that study subjects will benefit from the satisfaction that they are participating and contributing to SMA research.

The risk/benefit ratio is favorable since participation in the study is reasonably commensurate to routine care for an SMA subject over the course of one visit.

- 1. Limitations of the Study

Several limitations were considered in the design of the study.  Outlined below is how the study proposes to mitigate these limitations:

1. Biomarkers may vary by age and Type I subjects will be on the younger end of the age distribution.  The proposed solution is to control for age in all statistical models.
2. Based on the inclusion criterion for age, the study outcomes may not be generalization to children <2 or >12 years old. The primary outcome is not reliable in children less than 2 years of age. Any putative biomarkers identified in this effort would need to be validated in follow-on studies in a broader age distribution.
3. The sample size selected is based on number of specimens collected and it is possible that less than 100% of specimens collected will be analyzable. The study will allow for replacement of subjects to account for these specimens.
4. There is a potential that differences in standards of care between centers or regional differences (in diet or lifestyle) may have an impact on putative biomarker levels  It is unknown whether this has an effect prior to identification of a biomarker.  An ideal biomarker would be robust against effects of lifestyle and diet. The study design attempts to mitigate this potential effect by instituting site caps by cohort.
5. Recruitment of control children may be difficult as they do not have a direct or indirect benefit (other than siblings) from the study.  It is possible that this will be an issue; however the pool of control children is significantly larger than the SMA pool and therefore 20 control individuals is achievable.

# PROTOCOL ADMINISTRATION

- 1. Good Clinical Practices

The study will be conducted in accordance with the principles of Code of Federal Regulations, Title 45, Public Welfare Department of Health And Human Services, Part 46 Protection Of Human Subjects (revised June 23, 2005) (30), ICH Good Clinical Practice (CPMP/ICH/135/95; January 1997) and applicable regulatory requirements. Essential clinical documents will be maintained to demonstrate the validity of the study and the integrity of the data collected. Master files will be established at the beginning of the study, maintained for the duration of the study and retained according to the appropriate regulations (31).

- 1. Ethical Considerations

The study will be conducted in accordance with ethical principles founded in the Declaration of Helsinki. (32) The IRB/IEC will review all appropriate study documentation in order to safeguard the rights, safety and well being of the subjects. The study will only be conducted at sites where IRB/IEC approval has been obtained. The protocol, informed consents, advertisements (if applicable), written information given to the subjects, safety updates, annual progress reports and any revisions to these documents will be provided to the IRB/IEC by the investigator.

- 1. Protocol Compliance

The investigator will conduct the study in compliance with the protocol and will obtain approval by their local IRB/IEC. Modifications to the protocol should not be made without agreement of both the investigator and the Sponsor. Changes to the protocol will require written IRB/IEC approval prior to implementation, except when the modification is needed to eliminate an immediate hazard(s) to subjects or if the change(s) involves only logistical or administrative aspects of the study. The IRB/IEC may provide, if applicable regulatory authority (ies) permit, expedited review and approval/ favorable opinion for minor change(s) in ongoing studies that have the approval of the IRB/IEC.

When immediate deviation from the protocol is required, the investigator will contact the Sponsor, if the circumstances permit, to discuss the planned course of action. Any departures from the protocol must be fully documented in the source documentation.

- 1. Subject Confidentiality

In order to maintain subject confidentiality the study will take the following steps:

- Case report forms, study reports and communications will identify the subject by the assigned subject number. Participant names, initials, social security numbers or hospital ID numbers will not be used.
- The investigator will grant monitor(s) and auditor(s) from the Sponsor, the Coordinating Center, the IRB/IEC and regulatory authority(ies) access to the subject’s original medical records for verification of data gathered on the case report forms and auditing of the data collection process.
- All specimens collected will be labeled only with the date of collection, random barcode and study-specific subject ID number.
- The subject’s confidentiality will be maintained and will not be made publicly available to the extent permitted by the applicable laws and regulations.
- Shared datasets (see section 12.8 Publication and Use of Information) will not include patient identifiers, participant initials or dates of birth.
- At clinic sites, hard copy data collection forms will be stored in locked filed cabinets or locked rooms.
- Confidentiality of computerized data will be preserved by means of unique passwords and identification checks. Access to the ADEPT web-based data management system is password controlled and passwords must be changed every 90 days. The system includes an automatic timeout and will terminate the session automatically if not activity has occurred for 30 minutes.
  1. Specimen Retention

Specimens collected may be stored for up to 10 years following the completion of the study and will be destroyed after that time. Specimen destruction will be documented. In addition, specimens can be destroyed at any time at the subject’s or subject’s parents/guardians request.

- 1. Record Retention

An investigator is required to prepare and maintain adequate and accurate case histories that record all observations and other data pertinent to the investigation on each individual for both SMA and control cohorts in the investigation. Case histories include the case report forms and supporting data including, for example, signed and dated consent forms and medical records including, for example, progress notes of the physician, the individual's hospital chart(s), and the nurses' notes. The case history for each individual shall document that informed consent was obtained prior to participation in the study. An investigator shall retain records required to be maintained under this part for a period of 10 years following the study publication. Should investigators be unable to continue maintenance of subject files for the above duration, the Sponsor will assist in this regard.

- 1. Study Termination by the Sponsor

This study may be prematurely terminated if, in the opinion of the investigator or Sponsor, there is sufficient reasonable cause. Written notification documenting the reason for such a termination will be provided to the investigator or Sponsor.

Circumstances that may warrant termination include, but are not limited to:

- Determination of unexpected, significant or unacceptable risk to the subject.
- Failure to enter subjects at an acceptable rate.
- Insufficient adherence to protocol requirements.
- Insufficient complete and/or evaluable data.
  1. Publication and Use of Information

The Spinal Muscular Atrophy Foundation (SMA Foundation, “Sponsor”) is sponsoring this pilot study to stimulate the identification and development of biomarkers with the sensitivity and specificity for use as validated pharmacodynamic markers and/or surrogate endpoints. To that end, the SMA Foundation has agreed to make data generated during this study available to qualified investigators in the field after a period of confidentiality through a recognized data repository program. It is anticipated that this time period will coincide with the submission of the primary study-wide results manuscript as driven by the Protocol Committee and the sponsor. The shared data set will include de-identified subject data. Dates of birth and site location will not be included in the shared data set.

A Publications Committee appointed by the sponsor will review and approve all publications until the data is accessible to the public. No trial data may be presented or published without prior written approval of the Publications Committee. Final manuscripts prepared by members of the Publications Committee will be approved by the SMA Foundation before submission for publication. Detailed Publication Policies will be included in the Manual of Operations.

# REFERENCES

28. <http://www.cdc.gov/growthcharts/>

29. <http://www.drgreene.org/body.cfm?id=21&action=detail&ref=1616>

30. <http://www.hhs.gov/ohrp/humansubjects/guidance/45cfr46.htm>

31. <http://www.accessdata.fda.gov/scripts/cdrh/cfdocs/cfcfr/CFRSearch.cfm>

32. <http://www.wma.net/e/policy/b3.htm>

Signature Page

| **Pilot Study of Biomarkers** **for Spinal Muscular Atrophy**  **(BforSMA)** |
| --- |

The study will be conducted in accordance with the principles of Code of Federal Regulations, Title 45, Public Welfare Department Of Health And Human Services, Part 46 Protection Of Human Subjects (revised June 23, 2005), ICH Good Clinical Practice (CPMP/ICH/135/95; January 1997) and applicable regulatory requirements.

Every effort has been made to ensure that the contents of this protocol were complete and accurate at the time of issuance. If there are changes to be made to the protocol design or further key information becomes apparent after issue, it will be communicated to the Principal Investigator, the relevant IRB/IEC and regulatory authority(ies) as a formal amendment.

Authorized by:

_________________________ ____________________

Title Date

_________________________ ____________________

Title Date

_________________________ ____________________

Title Date
