# Supplementary material for: Evaluation of SMN Protein, Transcript, and Copy Number in the Biomarkers for Spinal Muscular Atrophy (BforSMA) Clinical Study
Source: PLoS One. 2012 Apr 27;7(4):e33572. doi: 10.1371/journal.pone.0033572 (PMC3338744; doi:10.1371/journal.pone.0033572)
Supplement: Table S1 — Effect of Age on (log) SMN Transcript Levels in SMA and Control Subjects. * Regression of age on SMN transcript levels, controlling for SMA diagnosis and Type. There were no significant interactions between age and diagnosis/type. (DOC) [file pone.0033572.s004.doc]

**Table S1.** Effect of Age on (log) SMN Transcript Levels in SMA and Control Subjects

|  | **Slope (per year)** | | | | **P-value*** |
| --- | --- | --- | --- | --- | --- |
|  | **SMA I** | **SMA II** | **SMA III** | **Controls** | **age** |
| **SMN-FL** | -0.03 | -0.02 | -0.03 | -0.10 | **0.008** |
| **SMN- Δ7** | -0.03 | 0.01 | -0.05 | -0.06 | 0.16 |
| **SMN-total** | -0.03 | 0.00 | -0.04 | -0.09 | **0.036** |
| **GAPDH** | -0.06 | 0.03 | -0.01 | -0.01 | 0.97 |
