# Supplementary material for: Evaluation of SMN Protein, Transcript, and Copy Number in the Biomarkers for Spinal Muscular Atrophy (BforSMA) Clinical Study
Source: PLoS One. 2012 Apr 27;7(4):e33572. doi: 10.1371/journal.pone.0033572 (PMC3338744; doi:10.1371/journal.pone.0033572)
Supplement: Table S2 — SMN Transcripts (log) and Protein (log) by SMA and Control Subjects: pair-wise comparisons. Analysis of covariance (ANCOVA) of SMA Type I vs. II vs. III vs. Control, controlling for age. The p-values presented here represent pair-wise post-hoc comparisons from the single model. GAPDH values are not shown as they remained unchanged. The mean and median values for transcript, protein and copy number data by SMA type are presented in Table 2. *SMN2 copy number values were not log transformed as they are ordinal values. (DOC) [file pone.0033572.s005.doc]

**Table S2. SMN Transcripts (log) and Protein (log)** by SMA and Control Subjects: pair-wise comparisons

|  | p-value | | | | | |
| --- | --- | --- | --- | --- | --- | --- |
|  | Within SMA Type comparisons | | | SMA Type vs Control | | |
|  | I vs. II | I vs. III | II vs. III | Type I | Type II | Type III |
| SMN-FL | **0.033** | **<0.001** | **0.020** | **<0.001** | **<0.001** | **<0.001** |
| SMN- Δ7 | **0.022** | **0.043** | 0.78 | 0.14 | **<0.001** | **<0.001** |
| SMN-total | **0.008** | **0.006** | 0.80 | **0.052** | 0.75 | 0.62 |
| SMN2-FL / SMN-Δ7 | 0.78 | 0.071 | **0.006** | **0.007** | **<0.001** | **0.015** |
| SMN protein | 0.12 | 0.10 | 0.90 | **<0.001** | **0.002** | **0.004** |
| SMN2 copy number* | **0.032** | **<0.001** | **0.013** | **0.007** | **<0.001** | **<0.001** |
